# Plasma proteome fingerprint in kidney diseases

**DOI:** 10.3389/fmolb.2024.1494779

**Published:** 2025-01-17

**Authors:** Kirill S. Nikolsky, Arthur T. Kopylov, Valeriya I. Nakhod, Natalia V. Potoldykova, Dmitry V. Enikeev, Tatiana V. Butkova, Liudmila I. Kulikova, Kristina A. Malsagova, Vladimir R. Rudnev, Denis V. Petrovskiy, Alexander A. Izotov, Anna L. Kaysheva

**Affiliations:** ^1^ Laboratory of Structural Proteomics, Institute of Biomedical Chemistry, Moscow, Russia; ^2^ Institute for Urology and Reproductive Health, I.M. Sechenov First Moscow State Medical University (Sechenov University), Moscow, Russia

**Keywords:** kidney diseases, kidney calculus, kidney cyst, cancer, tandem mass spectrometry, protein analysis, phosphorylation, candidate markers

## Abstract

**Introduction:**

Kidney diseases pose a serious healthcare problem because of their high prevalence, worsening of patients’ quality of life, and high mortality. Patients with kidney diseases are often asymptomatic until disease progression starts. Expensive renal replacement therapy options, such as dialysis or kidney transplant, are required for end-stage kidney disease. Early diagnosis of kidney pathology is crucial for slowing down or curbing further damage. This study aimed to analyze the features of the protein composition of blood plasma in patients with the most common kidney pathologies: kidney calculus, kidney cyst, and kidney cancer.

**Methods:**

The study involved 75 subjects. Proteins associated with kidney pathologies (CFB, SERPINA3, HPX, HRG, SERPING1, HBB, ORM2, and CP) were proposed. These proteins are important participants of complement and coagulation cascade activation and lipid metabolism.

**Results:**

The revealed phosphorylated proteoforms (CFB, C4A/C4B, F2, APOB, TTR, and NRAP) were identified. For them, modification sites were mapped on 3D protein models, and the potential role in formation of complexes with native partner proteins was assessed.

**Discussion:**

The study demonstrates that the selected kidney pathologies have a similar proteomic profile, and patients can be classified into kidney pathology groups with an accuracy of (70–80)%.

## 1 Introduction

Understanding the molecular foundations of disease development opens up new opportunities for designing novel diagnostic approaches as well as searching for candidate biomarkers and protein drug targets. The pathogenesis of multifactorial diseases is determined by changes in gene expression and, therefore, changes in the protein profile of biospecimens as well as the presence of modified (aberrant) proteoforms (Kopylov et al., 2023; Tikhonov et al., 2021; Nikolsky et al., 2023). Comparative case–control studies fail to provide insight into whether there are any differences between etiologically similar disease groups or determine specificity of the revealed candidate markers for segmentation of diseases of a certain organ. Implementation of research findings into medical practice is significantly impeded by low specificity of candidate biomarkers. In this context, conducting system research that focuses on the features of molecular profiles of etiologically similar diseases or diseases of one organ or tissue is rather relevant.

Our study addresses the most widespread kidney diseases. The comparison groups involved patients with kidney calculus, kidney cyst, and malignant kidney neoplasm. These pathologies are extremely common nowadays: up to 15% of global population are prone to the development of kidney calculus (Moftakhar et al., 2022) and at least 25%, simple kidney cysts (Garfield and Leslie, 2024). The annual incidence of kidney cancer is 400,000 new cases (Cirillo et al., 2024). There is heterogeneity in global epidemiology of these diseases: their highest prevalence is typical of Europe and North America.

These diseases have a largely similar clinical presentation (Table 1), thus hampering timely and accurate clinical diagnosis. Malignant transformation of kidney cysts (Tseng, 2015; Lu et al., 2021; Lai et al., 2019) and disease mimicry [e.g., renal cell carcinoma mimics kidney cyst (Jiang et al., 2015; Yu et al., 2017)] are possible.

**TABLE 1:** Comparison of clinical presentation of the development of kidney pathology.

Parameter	Kidney calculus	Kidney cyst	Kidney cancer
Disease and risk factor control			
Symptoms	• severe pain on either side of lower back • pain or stomach ache • hematuria • nausea or vomiting • fever and chills	• pain in the side, stomach or low back • hematuria • fever • changes in urination habits	• pain or dull ache in the side or lower back • hematuria • fever • changes in urination habits • rapid, unexplained weight loss
Diagnosis	Ultrasound computed tomography (USCT) or urography	Computed tomography (CT) Magnetic resonance imaging (MRI) Contrast-enhanced ultrasound (CEUS)	USCT or contrast-enhanced MRI; Histological diagnosis
BMI	Overweight (Moftakhar et al., 2022)	n/d	Obesity (Gray and Harris, 2019)
Diabetes mellitus	Yes (Moftakhar et al., 2022)	Yes (Wei et al., 2020)	Yes (Tseng, 2015)
Hematuria	Yes (Nagendra et al., 2023)	Yes (Boo et al., 2022)	No (Takeuchi et al., 2021)
Hypertension	Yes (Moftakhar et al., 2022)	Yes (Kim et al., 2014)	Yes (Gray and Harris, 2019)
Cigarette smoking	Yes (Moftakhar et al., 2022)	Yes (Sousa et al., 2021)	Yes (Gray and Harris, 2019)
Adults aged >40 years	Yes (Moftakhar et al., 2022)	Yes (Wei et al., 2020)	Yes (Tseng, 2015)
Sex	Yes	n/d	Yes (Ferlay et al., 2018; Moch et al., 2016)
Hereditary predisposition	Yes (Howles and Thakker, 2020; Singh et al., 2022)	Yes (Satariano et al., 2024; Park et al., 2021; Dillman et al., 2017)	Yes (Truong et al., 2022; Shuch and Zhang, 2018; Bratslavsky et al., 2021; Yanus et al., 2024)
Molecular factors			
Creatinine (serum)	Elevated (Katwal et al., 2022; Shastri et al., 2023; Shen et al., 2022; Pan et al., 2024)	n/d	Elevated (Liu et al., 2020; Zhang and Bu, 2022)
Urea (serum)	Elevated (Katwal et al., 2022)	n/d	Elevated (Sun et al., 2021; Bai et al., 2021; Li et al., 2019)
Uric acid (serum)	Elevated (Kc and Leslie, 2024; Tung et al., 2024; Alelign and Petros, 2018)	Elevated (Abbiss et al., 2019)	Elevated (Allegrini et al., 2022; Dai et al., 2020)
Calcium (ionized or total)	Elevated (Alelign and Petros, 2018)	Elevated (Mangolini et al., 2016)	Hyperelevated (Vakiti et al., 2024)
Albuminuria	Elevated (Alelign and Petros, 2018; Brewin et al., 2021)	Elevated (Boo et al., 2022)	Elevated (Luo et al., 2023a; Luo et al., 2023b; Mok et al., 2020)
C-reactive protein	Elevated (Shoag and Eisner, 2014)	Elevated (Bergmann et al., 2018)	Elevated (Zhou et al., 2015)
Lactate dehydrogenase (LDH)	Elevated (Devi et al., 2023; Ding et al., 2021; Xiao et al., 2023)	n/d	Elevated (Claps et al., 2022; Wu et al., 2021; Mishra and Banerjee, 2019)
Interleukin-1 (IL-1)	Association (Xiao et al., 2022)	Association (Yang et al., 2019)	Association (Sindhughosa and Pranamartha, 2017)
PKD1/PKD2	n/d	Association (Yang et al., 2023; Al-Hamed et al., 2019)	Association (Meguro et al., 2023)
Alkaline phosphatase	Elevated (Palsson et al., 2019; Zhu et al., 2023)	n/d	Elevated (Stebbing et al., 2014; Stanford and Bottini, 2023)

Symptoms, clinical presentation, risk factors and even candidate molecular factors are often similar for kidney pathologies. Today, searching for markers or an ensemble of markers that would meet the clinical requirements for specificity, accuracy and sensitivity in the context of early diagnosis of kidney diseases is still ongoing. Thus, blood biochemistry test is recommended for suspected kidney cancer, with special attention paid to albumin blood level, the erythrocyte sedimentation rate, lactate dehydrogenase (LDH) and alkaline phosphatase activities, as well as blood levels of ionized and total calcium. These serologic factors are not specific to oncopathology (Table 1) but may affect the choice of treatment approach, as well as diagnostic and secondary prophylaxis options (Chen et al., 2017).

Elevated levels of reactive oxygen species, lipid peroxidation products, proinflammatory cytokines, and proangiogenic factors are typically detected in patients with kidney calculus (Wigner et al., 2021). Parameters recommended to be measured in these patients include the levels of cations (calcium, potassium, and sodium), C-reactive protein, creatinine, and albumin (EAU, 2024b; Vitale et al., 2008).

In patients with kidney cyst, blood chemistry parameters often remain normal if the cyst neither exerts pressure on adjacent tissue nor disturbs the renal function. For this disease, there are very few publications addressing the biochemical markers in blood of patients diagnosed with kidney cyst.

A proteomic analysis of blood samples collected from patients with verified diagnosis of kidney calculus, kidney cyst, or kidney cancer was conducted to identify proteins making the greatest contribution to segmentation of comparison groups. Phosphorylated proteoforms specific to groups of patients with kidney diseases were analyzed, and potential signaling pathways for pathology development were identified.

## 2 Materials and methods

### 2.1 Ethical consideration

Patients and healthy volunteers provided written consent to participate in the study, which was approved by the Local Ethics Committee of the Sechenov University (protocol No. 10–19 dated 17 July 2019). All handlings and use of material were provided according to the WMA Declaration of Helsinki on Ethical Principles for Medical Research Involving Human Subjects (the revision approved in Fortaleza, 2013). All the participants were aware of the research purpose. Informed consent was obtained from all the participants.

### 2.2 Participants

The study involved 75 subjects, including 51 patients having a verified diagnosis of a urologic disease: kidney calculus, kidney cyst, and kidney cancer, as well as 24 conditionally healthy subjects (control group). The inclusion criteria for the group of participants diagnosed with a urological disease were as follows:

– patient’s age ≥18 years;
– ICD 10 code of the underlying disease: N20.0 “Calculus of kidney”, N28.1 “Cyst of kidney, acquired”, C64 “Malignant neoplasm of kidney, except renal pelvis”.

The non-inclusion criteria were as follows:

– patients diagnosed with cancer of other organs and systems;
– psychoactive substance and alcohol abuse;
– pregnant or breastfeeding women.

The kidney disease (KD) group consisted of patients having kidney calculus and kidney cyst. The final diagnosis in the kidney cancer group (KC) was made based on multislice computed tomography (MSCT) and histologic examination.

Triple-phase helical computed tomography of the abdomen and the peritoneal cavity (kidneys) using the bolus-tracking technique was conducted regardless of disease stage in order to differentiate between benign and malignant tumors. Scans were acquired before and after administration of a contrast agent to assess its accumulation. Contrast enhancement of the neoplasm was determined by comparing the Hounsfield scale parameters (in Hounsfield units, HU) before and after administration of the contrast agent. Changes in contrast by at least 15 HU were regarded as contrast enhancement and viewed as a functionally active tumor region (EAU, 2024a). Malignancy and tumor grade were verified by postoperative histological examination.

Cystic structures were detected and classified using the Bosniak system. The group of patients with kidney disease comprised patients with Bosniak I or II cysts as these cystic structures are benign.

The number of subjects in the kidney disease (KD) group was 25; in the kidney cancer (KC) group, 26; and in the healthy (CNTR) group, 24 (Table 2). The comparison groups were matched with respect to the number of subjects and their age, sex, and height distribution. Differences in these parameters between comparison groups were non-significant among males and females (p ⩾ 0.05).

**TABLE 2:** Anthropometric characteristics of the study participants.

Parameter	Kidney disease (KD)		Kidney cancer (KC)		Healthy group (CNTR)	
ICD-10 code of the underlying disease	N20.0 “Calculus of kidney”; N28.1 “Cyst of kidney, acquired”		C64 “Malignant neoplasm of kidney, except renal pelvis”		–	–
Sex	female	male	female	male	female	male
Number of participants (n)	15	10	11	15	12	12
Age (years)	59 ± 11.83 (*p* = 0.47)	58.5 ± 13.74 (*p* = 0.6)	57 ± 7.88 (*p* = 0.72)	60 ± 5.47 (*p* = 0.6)	60.5 ± 3.7	59.5 ± 2.19
Weight (kg)	76 ± 12.85 (*p* = 0.05)	86.5 ± 12.73 (*p* = 0.001)	98 ± 12.37^a^ (*p* = 0.002)	95 ± 9.97^a^ (*p* = 0.0003)	68.5 ± 5.92	68.5 ± 7.35
Height (cm)	164 ± 7.17 (*p* = 0.32)	179.5 ± 4.84 (*p* = 0.05)	164 ± 3.03^a^ (*p* = 0.38)	182 ± 6.88^a^ (*p* = 0.05)	171.5 ± 9.21	173.5 ± 6.89
BMI (kg/m^2^)	28.3 ± 5.18 (*p* = 0.01)	25.43 ± 3.86 (*p* = 0.001)	35.9 ± 4.72^a^ (*p* = 0.001)	27.8 ± 3.65^a^ (*p* = 0.0002)	23.51 ± 1.57	22.89 ± 1.66

^a^ – according to the available data; p-value (Benjamini–Hochberg adjusted) are provided with respect to the values of the control (comparison) group of patients of the respective sex.

The body mass index (BMI) parameters come under notice, which differed significantly in the KD and KC groups compared to patients in the control group. The mean BMI among all the female urologic patients was 32.1 ± 4.95 kg/m^2^, which corresponded to overweight or class 1 obesity. Among male patients, this parameter was 26.62 ± 3.76 kg/m^2^, mostly corresponding to normal body weight or overweight. Patients diagnosed with kidney cyst or kidney calculus had BMI = 28.3 ± 5.18 kg/m^2^ (females) and 25.43 ± 3.86 kg/m^2^ (males). For study subjects diagnosed with kidney cancer, BMI was 35.9 ± 4.72 kg/m^2^ among females (corresponding to class 1 and 2 obesity) and 27.8 ± 3.65 kg/m^2^ among males (mostly overweight and class 1 obesity). The control group consisted of conditionally healthy subjects with BMI lying in the normal range.

### 2.3 Blood sample collection

Blood samples in study subjects were collected strictly after fasting, at 8.00–10.00 a.m., in a clinical diagnostic laboratory. For proteomic analysis, blood samples were collected from the cubital vein into vacutainer tubes containing 3.8% sodium citrate anticoagulant (Improvacuter, Guangzhou Improve Medical Instruments Co., Ltd., Guangzhou, China). The samples were centrifuged at 3,000 rpm during 6 min at room temperature. Each plasma sample (500 µL) was collected into two dry Eppendorf polypropylene microtubes, frozen, and stored at −80°C until analysis.

### 2.4 Blood chemistry test

Blood chemistry tests were conducted on a Torus 1,200 semi-automatic chemistry analyzer (Moscow, Russia). Reaction mixtures were prepared in accordance with the manufacturer’s instructions. The measurements were performed using the kinetic method, the endpoint method, and fixed time. Quality control was performed by measuring control “normal” (TruLab N) and “pathological” (TruLab P) sera (DiaSys Diagnostic Systems GmbH, Germany). The reference values from the application sheets are summarized in Table 3.

**TABLE 3:** Blood chemistry parameters of venous blood and their reference values.

Parameter	Abbreviation	Reference values		Units
		Male	Female	
Alanine aminotransferase	ALT	10-40 (Eliseev, 2023)	7–35	U/L
Aspartate aminotransferase	AST	<40 (Eliseev, 2023)	<40	U/L
Alkaline phosphatase	ALP	<270 (Eliseev, 2023)	<240	U/L
Albumin	ALB	35–50		g/L
Triglycerides	TRIG	40–49 years 0.63–3.37 >50 years 0.70–3.25 (Eliseev, 2023)	40–49 years 0.50–2.10 >50 years 0.62–2.79	mmol/L
Creatinine	Cr	>60 years 71–115 (Eliseev, 2023)	>60 years 53–106	μmol/L
Glucose	Glu	>60 years 4.6–6.4		μmol/L

### 2.5 Preliminary preparation of blood plasma for HPLC-MS/MS analysis

Preliminary preparation of blood plasma for proteomic analysis was thoroughly described in Stepanov et al. (2023). The protein fraction was precipitated with methanol (JT Baker, Landsmeer, the Netherlands). Alkylation was performed in the presence of 2% 4-vinylpyridine solution (Aldrich, Gillingham, United Kingdom) in 30% isopropanol solution (Fisher Chemical, Laughborough, United Kingdom). Trypsinolysis (∼400 ng trypsin per sample, Promega, Madison, Wisconsin, United States) involved two steps and was performed in 75 mM triethylammonium bicarbonate buffer (pH 8.2). The hydrolysates were vacuum dried before storage (Concentrator Plus, Eppendorf, Hamburg, Germany). Before mass spectroscopy measurements, the dry residue was re-dissolved using 0.1% formic acid (Acros Organics, Geel, Belgium).

### 2.6 Mass spectrometry analysis

Spectra were acquired on a Xevo™ G2-XS Q-TOF quadrupole time-of-flight mass spectrometer (Waters, Wilmslow, United Kingdom) in the positive ionization mode within a normal dynamic range of masses. The capillary voltage was 3 kV; the cone voltage was 67 V. The drying gas flow rate was 680 L/min; the focusing gas velocity was 50 L/min. The source temperature was set at 150°C; desolvation temperature was set at 350°C. Ions were surveyed in the data-independent acquisition mode (SONAR). The full MS scanning was performed in a range of 300–1,300 m/z with a low-energy CID fragmentation at 6 eV, followed by scanning in the SONAR mode with quadrupole mass start at m/z = 178 and quadrupole mass stop at m/z = 1,287 with a 22 Da mass window and high-energy CID fragmentation range from 15 to 37 eV. The total time for a complete scan cycle was set at 0.418 s. Mass correction using leucine–enkephalin (m/z = 556.2771, z = 1+) injected every 30 s at a concentration of 50 pg/mL was active at scanning times from 0.5 to 59 min.

Chromatographic separation was performed on an Acquity™ UPLC H-Class Plus chromatography system (Waters, United Kingdom) using a BEHC18 column (1.7 µm particle size, geometry 2.1 × 50 mm; Waters, United Kingdom) at 40°C and a flow rate of 0.3 mL/min in gradient mobile phases A (water) and B (acetonitrile) supplemented with 0.1% formic acid and 0.03% acetic acid. The following elution gradient was applied: 0–1.5 min, 3% B; up to 26.5 min, 19% B; up to 42 min, 32% B, and up to 43.5 min, 97% B; the column was then exposed to the isocratic mode up to 47.5 min and 3% B at 49 min. The post-run column equilibration lasted 6 min.

The obtained data were analyzed using the PLGS (Protein Lynx Global Server, version 3.0.3, Waters, United Kingdom) software employing the UniProt KB database with preset parameters for the SONAR/MS^E^ scanning mode (Stepanov et al., 2023). The data were processed using the Apex 3D mode with a lock-mass correction of leucine–enkephalin (m/z = 556.2771^1+^) within 0.2 Da detection window. Low-energy and high-energy intensity thresholds were adjusted to 60 and 10 counts, respectively, between 0.5 and 49 min of the acquired data. Spectral data after processing were browsed against Uni Prot KB (release September 2023) protein sequences with a concatenated reverse-sequences database. Ions were surveyed with a peptide tolerance of ±25 ppm and fragment ion tolerance of ±75 ppm using at least three fragment ion matches per peptide and at least two peptide matches per protein after digestion with trypsin with a maximum of one missed cleavage. S-pyridylethylation was applied as a fixed type of modification, while S, T and Y phosphorylation (SEP, TPO and TPR) was applied as a variable modification. Peptide and protein identifications were percolated using the 2% false discovery rate. The mass spectrometry proteomics data have been deposited to the ProteomeXchange Consortium via the PRIDE (Perez-Riverol et al., 2021) partner repository with the dataset identifier PXD051799 “Kidney disease proteome research” (Reviewer account details: Username: reviewer_pxd051799@ebi.ac.uk; Password: R858MIv3).

### 2.7 Data analysis

PSMs detected with PLGS were processed with two steps:

1. Filtering: only samples with more than 70 unique proteins (by UniProt ID) identified in sample were chosen for next steps;
2. Imputation: missing concentration values for PSMs were predicted using ordinary least squares (OLS) linear regression based on the available intensity values.

Differentially expressed proteins were identified for a sample (N = 57) filtered according to the following criteria:

1. Protein is found in ≥50% samples within a group;
2. Concentration was imputed in ≤50% protein samples within a group.

The fold change value was calculated as follows:

If $C_{ctrl}>C_{test}$ , then $fCh=\frac{C_{test}}{C_{ctrl}}$ ; else $fCh=\frac{C_{ctrl}}{C_{test}}*\left(-1\right)$ , where C_cntr_ is the median protein concentration in the control group; C_test_ is the median protein concentration in the comparison group; fCh is the fold change.

The p-value was determined by Mann–Whitney U rank test (Mann and Whitney, 1947) in the scipy. stats package (SciPy, 2024b). False discovery control was performed employing the Benjamini–Hochberg procedure (Benjamini and Hochberg, 1995) also using the scipy. stats package (SciPy, 2024a).

Principal component analysis (PCA) and Sparse partial least-squares discriminant analysis (sPLS-DA) with 0.95 ellipse confidence were conducted using the MixOmics software package for R programming language (mixOmics, 2024) based on the examples of using these methods (sPLSDA SRBCT Case Study, 2024; mixOmics Vignette, 2024) for a group of proteins (N = 104) that were identified in no less than 20 samples.

The acceptance criteria for protein identification were based on the Human Proteome Project Mass Spectrometry Data Interpretation Guidelines 3.0 (Malsagova et al., 2024): a candidate feature in the proteomic data had to meet the unicity criterion, meaning that certain proteins had to be covered by more than one unique protein-specific peptide without interference from any other protein.

## 3 Results

### 3.1 Analysis of blood chemistry data

In all study subjects, blood chemistry parameters were either normal or lay within the lower limit of the normal range. Slight decline in albumin level as well as reduced creatinine level and alanine aminotransferase activity were observed in the group of patients with kidney cancer. Blood glucose, triglyceride, and albumin levels were either normal or slightly decreased in the group of conditionally healthy subjects (Table 4).

**TABLE 4:** Blood chemistry parameters of study subjects.

Parameter	Kidney disease (KD)^a^	Kidney cancer (KC)^a^	Healthy group (CNTR)^a^
Glu (μmol/L)	5.2 ± 1.96 (*p* = 0.002)	5.25 ± 1.29 (*p* = 0.002)	3.8 ± 1.39
TRIG (μmol/L)	1.16 ± 0.55 (p= 0.000001)	0.98 ± 1.72 (*p* = 0.00003)	0.32 ± 0.24
ALB (g/L)	36 ± 13.18 (*p* = 0.86)	35.5 ± 9.37 (*p* = 0.86)	35 ± 7.81
ALT (U/L)	9.5 ± 4.22	4.0 ± 4.3	n/d
AST (U/L)	10.05 ± 4.47	10.57 ± 3.44	n/d
ALP (U/L)	132.5 ± 29.85	171 ± 75.62	n/d
Cr (μmol/L)	67 ± 18.62	64 ± 16.9	n/d

^a^ – according to the available data; p-value (Benjamini–Hochberg adjusted) are provided with respect to the values of the control (comparison) group of patients of the respective sex.

For all thee selected parameters, the resulting values lie within the normal range. However, triglyceride and glucose levels in the KD and KC groups are significantly elevated compared to the CNTR group. The normal ranges of blood chemistry parameters in the KC group are possibly caused by the fact that in almost all the cases, there was a malignant tumor structure sized ≤7 cm, correlating with stage T1 according to the UICC TNM Classification of Malignant Tumors 2017, eighth edition (Brierley et al., 2017).

### 3.2 Plasma proteins

**FIGURE 1:**
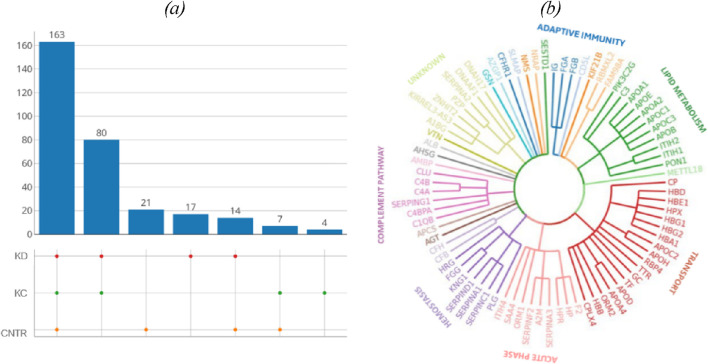
**(A)** UpSet plots of proteins shared among the studied groups. Intersection size denotes the number of proteins unique to a certain comparison group (a single point in the diagram) and shows the number of proteins shared by at least two comparison groups (dots of one sector). **(B)** The phylogenetic plot showing the distribution of biological processes in which proteins shared by the comparison groups are involved. Radial ribs correspond to molecular functions; ribs groups denote the biological processes according to their color. Each radial rib corresponds to a specific protein and is denoted with a respective protein-coding gene.

A total of 644 proteins were identified in the analyzed samples; 163 of them were shared among comparison groups (Figure 1A). Only few proteins were specific to groups of patients with kidney pathologies KC or KD. For this reason, no analysis of group-specific proteins was conducted.

The identified proteins shared by the analyzed comparison groups are expectedly involved in important biological processes (Figure 1B): classical antibody-mediated complement activation (R-HSA-173623, n = 46); FCGR activation (R-HSA-2029481, n = 45); binding and uptake of ligands by scavenger receptors (R-HSA-2173782, n = 54). A full list of shared proteins is provided in Supplementary Table S1.

Table 5 summarizes proteins whose blood level (fold change) differs for the selected comparison groups (KD and KC) with respect to control. The blood levels of proteins involved in immune response are simultaneously elevated in two comparison groups (kidney disease (KD) and kidney cancer (KC)) with respect to the control group. Thus, the blood level of complement factor B (P00751) in patients with kidney cancer increased 1.7-fold (р = 0.004); similar elevation was observed in patients in the kidney disease group (1.4-fold; *p* = 0.13). Furthermore, the blood levels of such proteins as alpha-1-acid glycoprotein 2 (P19652) (р = 0.02) and ceruloplasmin (P00450) (р = 0.04) in patients in the kidney disease and kidney cancer groups were also simultaneously increased 1.4-fold and 1.3-fold, respectively. The maximum change in protein level was observed for hemoglobin subunit beta (P68871): its level increased more than twofold (*p* = 0.08) in the KD group and more than fourfold, in the KC group (р = 0.0008).

**TABLE 5:** Most significantly altered proteins measured in the study cohorts (estimated as fold changes; |FC| > 1.5, Benjamini–Hochberg adjusted p-value cut-off *p* < 0.05 for one or both comparison groups).

UniProt ID	Gene	Protein	Pathway description	KD		KC	
				Fold changes	Adj. p-value	Fold changes	Adj. p-value
P00751	CFB	Complement factor B	Complement activation, alternative pathway	1.4	0.13	1.7	0.004
P01011	SERPINA3	Alpha-1-antichymotrypsin	Acute-phase response	1.5	0.045	1.4	0.05
P02790	HPX	Hemopexin	Positive regulation of immunoglobulin production	1.5	5.6 × 10^−5^	1.4	0.0003
P04196	HRG	Histidine-rich glycoprotein	Positive regulation of immune response to tumor cell	1.4	0.09	1.7	0.02
P05155	SERPING1	Plasma protease C1 inhibitor	Complement activation, classical pathway	1.5	0.003	–	–
P68871	HBB	Hemoglobin subunit beta	Oxygen transport	2.1	0.08	4.1	0.0008
P19652	ORM2	Alpha-1-acid glycoprotein 2	Acute-phase response	1.4	0.02	1.4	0.02
P00450	CP	Ceruloplasmin	Iron ion transport	1.4	0.04	1.3	0.04
P01825	IGHV4-59	Ig	Immunoglobulin-mediated immune response	1.6	0.005	1.5	0.01

**FIGURE 2:**
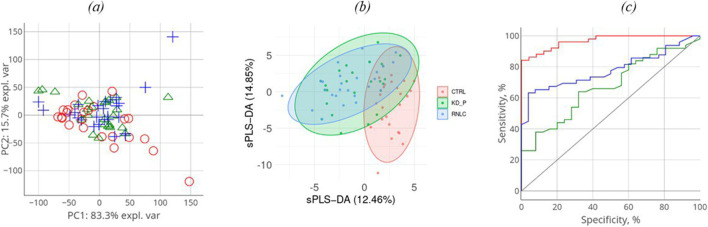
Discriminant analysis of studied cohorts: principal component analysis, PCA **(A)**. PCA indicates the intersample variance: the closer points lie in PCA, the more similar the proteomic profiles of the respective samples are. On each axis, the percentage corresponds to the total variance between all the points (PC1 = 83%, PC2 = 16%). Sparse partial least-squares discriminant analysis (sPLS-DA) for proteome **(B)** data type with 0.95 ellipse confidence level. sPLS-DA allows one to choose informative variables for segmentation of comparison groups. The designed score scattering plots show the relationship between the control group and patients (KD, KC), and the degree of variations that were explained by each component consisting of PC1 = 12.5% and PC2 = 14.9% for proteomic data **(C)** One of the most common metrics, Receiver Operating Characteristic Area Under the Curve ROC AUC, allows one to estimate quality of comparison group classification. The OX axis shows specificity (%), while the OY axis shows sensitivity (%).

The comparison groups could not be segmented by principal component analysis (PCA): the proteomic profiles were found to densely overlap (Figure 2A). For achieving discrimination, we performed selection and classification of descriptors using sparse partial least-squares discriminant analysis (sPLS-DA) (Figure 2B). The 12.5% and 15% variance in the consolidated proteome was interpreted by discriminant analysis (Figure 2B). Figure 2B demonstrates that the proteomic profiles corresponding to the control group differ to the maximum extent from the group of patients having a pathology. Meanwhile, the profiles of the KD and KC groups overlap as indicated by the large area of proteomic profile overlaps. Figure c shows that in general, the analysis provides a fair result of data segmentation into comparison groups (70%–80%).

**FIGURE 3:**
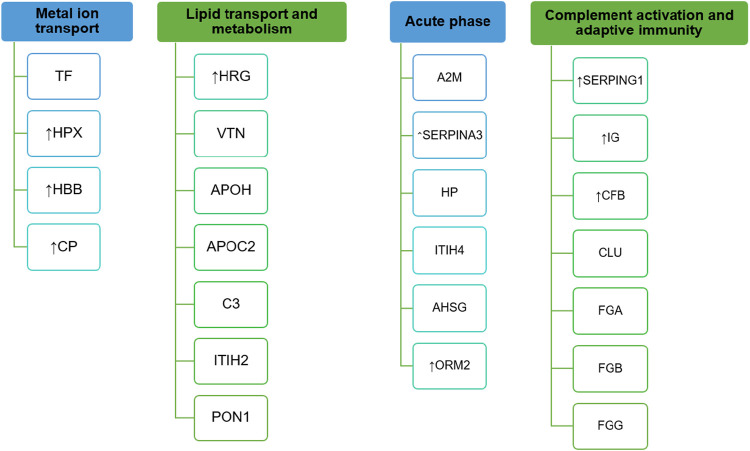
Names of the genes encoding proteins contributing most to segmentation of comparison groups clustered with respect to shared biological processes. Arrows show DEPs (*p* < 0.05).

In all three images in Figure 2A–C, the values referring to the control group, the KD group, and the KC group are shown in red, blue, and green, respectively. The absolute weights of the sparse loading vectors for each sPLS-DA dimension in the data set are presented in Supplementary Table S2; Figure 3 shows proteins with the greatest weights, including those whose level varied significantly.

Proteins making the greatest contribution to segmentation of comparison groups can either be classified into the four key biological processes, including lipid transport and metabolism, complement system activation, iron and heme transport, and implementation of adaptive immunity or are acute phase proteins (Figure 3).

### 3.3 Phosphorylated proteoforms

Post-translational modifications (PTMs) often affect protein function (Nikolsky et al., 2023). In this study, we analyzed the possible phosphorylated proteoforms, one of the most common native protein modifications. Table 6 lists the results of searching for phosphorylated proteoforms, including those carrying phosphorylated serine (SEP), phosphorylated threonine (TPO) and phosphorylated tyrosine (PTR).

**TABLE 6:** Proteins carrying a modifying moiety detected in the analyzed blood samples.

UniProt ID	Protein(s)	Pathways	Sequence variants	PTM loci	CNTR	KC	KD
P04114	Apolipoprotein B-100	Post-translational protein phosphorylation	VKYTLNKNSLK YTLNKNSLK	1287-PTR 1288-TPO	1	5	3
P00751	Complement factor B	Alternative complement activation	LEDSVTYHCSR VGSQYRLEDSVTYHCSR	∼183–194	1	4	5
P0C0L5 P0C0L4	Complement C4-B Complement C4-A	Activation of C3 and C5	TTNIQGINLLFSSRR GPEVQLVAHSPWLKDSLSRTTNIQGINLLFSSR DSLSRTTNIQGINLLFSSR	123–138	0	0	2
			DFALLSLQVPLK	86-SER	0	2	1
P02766	Transthyretin	Neutrophil degranulation	LLLLCLAGLVFVSEAGPTGTGESK	25-TPO	0	4	1
Q86VF7	Nebulin-related-anchoring protein	Ion binding	KKLHEYTVLPEDMK KLHEYTVLPEDMKTQWAK	659-PTR 660-TPO	3	0	0
P00734	Prothrombin	Regulation of the complement cascade	GHVNITR GHVNITRSGIECQLWRSR	123-TPO	1	3	3

Pathways–according to the Reactome data; Sequence variants–amino acid sequence variants that were detected in biosamples; PTM, Loci–the localization region or an accurate coordinate of PTM.

Table 6 shows that the phosphorylated proteoform of apolipoprotein B-100 was observed in eight blood biosamples in the KC and KD groups. Such proteoforms as complement factor B (n = 9), prothrombin (n = 6), and transthyretin (n = 5) are also more common in groups with a kidney pathology compared to the control group. Observation of these phosphorylated proteoforms in the investigated blood samples is interesting in terms of their abundance in the comparison groups. Meanwhile, these results do not provide information enabling precise classification of each group of patients with a pathology (KC and KD): occurrence of PTM-containing proteoforms is approximately identical in both groups, being indicative of the synergistic pathogenesis of the selected groups of urologic diseases (kidney calculus, kidney cyst, and kidney cancer). The results also suggest that this study needs to be continued using a significantly larger number of study subjects (patients having a kidney pathology (KC, KD) and control group (CNTR).

Analysis of the location of a modifying moiety with respect to the interface of binding to native partner proteins (the Interface column) helps assess the potential effect of a PTM on protein structure and functions. Table 7 lists the data on the topology and geometrical parameters of the identified proteoforms. Experimentally determined 3D structures in Protein data bank, including those forming complexes with partner proteins, were identified for four proteins listed in Table 7. For these proteins, it was found that the region carrying modified amino acid residues is located on the protein globule surface and is accessible for interacting with a partner protein. In three cases, the regions carrying modified amino acid residues are located directly at the interface of binding to the partner protein or near the binding site (see Table 7, K_SASA_ ≤ 1). The K_SASA_ parameter characterizes changes in the solvent-accessible surface area for a peptide in the protein chain and the protein–protein complex. This parameter indirectly indicates the location of peptide (in our case, carrying a modifying moiety) with respect to the interface of binding to the partner protein.

**TABLE 7:** Structural characterization of the phosphorylation sites in proteins with annotated 3D structures according to the PDB and AlphaFold databases.

Uniprot ID	Peptide seq	Name	Gene	Domain	Motif	Partners	PDB/PDB_cmp_	Location	K_SASA_	HB	HP	Interface
P00751	LEDSVTYHCSR	Complement factor B	CFB	Ribbon	β-hairpin	Q91132: Cobra venom factor P00746: Complement factor D P01024: Complement C3 P27918: Properdin	10/15	Protein globule surface	0.6 ± 0.05	11 ± 2	8 ± 1	In
P0C0L5	TTNIQGINLLFSSR	Complement C4-B	C4B	Sandwich	β-hairpin	P0C0L4: Complement C4-A	2/2	Protein globule surface	0.5 ± 0.01	14 ± 1	29 ± 0	In
	DFALLSLQVPLK						2/2	Protein globule surface	1.0 ± 0.06	2 ± 0	42 ± 1	Near
P0C0L4	TTNIQGINLLFSSR	Complement C4-A	C4A	Sandwich		O00187: Mannan-binding lectin serine protease 2	2/5	Protein globule surface	0.8 ± 0.2	17 ± 1	30 ± 1	In
	DFALLSLQVPLK						2/5	Protein globule surface	1 ± 0	2 ± 0	44 ± 1	Out
P00734	GHVNITR	Prothrombin	F2	Beta Barrel	Irregular domain	P00742: Coagulation factor X P12259: Coagulation factor V	1/8	Protein globule surface	1 ± 0	7 ± 2	11 ± 2	Out
P04114	PTR-TLNKNSLK Y-TPO-LNKNSLK	Apolipoprotein B-100	APOB	n/d	n/d	Q8NBP7: proprotein convertase subtilisin/kexin type 9 (Sun et al., 2012) P55157: microsomal triglyceride transfer protein large subunit (Walsh et al., 2015) Q9Y679: Lipid droplet-regulating VLDL assembly factor AUP1 (Zhang et al., 2017a)	–	–	–	–	–	–
P02766	LLLLCLAGLVFVSEAGP-TPO-GTGESK	Transthyretin	TTR	n/d	n/d	Q15109: Advanced glycosylation end product-specific receptor P05067: Amyloid-beta precursor protein (according to the IntAct database)	–	–	–	–	–	–
Q86VF7	KLHEYTVLPEDMK	Nebulin-related-anchoring protein	NRAP	n/d	n/d	P35609: Alpha-actinin-2 P50461: Cysteine and glycine-rich protein 3 (according to the IntAct database)	–	–	–	–	–	–

Functional domain identifier according to CATH v.4.3 (https://www.cathdb.info/); Motif–a structural motif where the PTM-carrying peptide is located; CATH v.4.3 (https://www.cathdb.info/); Motif–a structural motif where the PTM-carrying peptide is located; PDB–the number of protein structures in PDB; PDB_cmp_–the number of structures in PDB with this peptide containing protein–protein complexes; localization–mapping of the PTM-carrying peptide to the 3D structure of the protein; the coefficient of participation in binding site K_SASA_ is the ratio between SASA of the peptide in the complex and SASA of the peptide in the protein chain. It indirectly characterizes the location of the PTM-carrying peptide with respect to the interface of binding to the partner protein. K_SASA_ <1 is indicative of participation in the interface with the partner protein; K_SASA_ ≥1 means that the peptide lies outside the binding site; HB is the estimated number of hydrogen bonds between the peptide; Interface is the estimated location of the PTM-carrying peptide with respect to the interface of binding with the partner protein. IntAct database, release 246 (https://www.ebi.ac.uk/intact/home).

**FIGURE 4:**
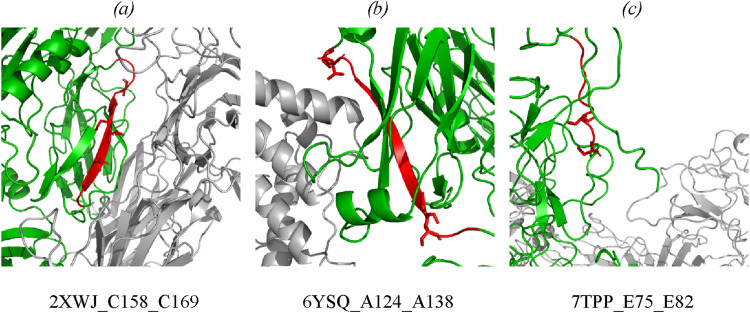
Mapping of the identified phosphorylated peptides in protein–protein complexes: **(A)** CFB in complex with complement C4a (PDB: 2XWJ); **(B)** complement C4b in complex with complement C4a (PDB: 6YSQ); **(C)** prothrombin in complex with activated factor Xa and coagulation factor Va (PDB: 7TPP). Modified proteins are shown in green; partners are shown in gray; detected peptides are shown in red; side chains of phosphorylated residues are shown as stick representation. Full data are available in the Supplementary Tables S3, S4.

The type of structural motif of the protein comprising the peptide carrying a modifying moiety was identified in each case. Thus, for CFB complexes, the partner of complement factor C4a (PDB ID 2XWJ) modified peptide is located in the dense β-hairpin of the beta-containing ribbon functional domain (Figure 4A). In proteins of different organisms, this domain type is involved in formation of protein–protein and protein–nucleic acid complexes (e.g., in zinc finger proteins, where the ribbon domain is stabilized by coordination bonds with a zinc cation) (Li et al., 2022). The K_SASA_ parameter of the CFB/C4a complexes is 0.6, indicating that the revealed CFB peptide carrying a modifying moiety is engaged in organization of the binding interface of the complex. Other members involved in activation of the complement cascade C4a/C4b also form complexes with partner proteins (PDB ID 6YSQ, Figure 4B). The identified modified peptides in the complexes are a part of β-hairpins of the sandwich functional domain. The sandwich functional domain in proteins is also an essential structural participant of protein complex formation (Nikolsky et al., 2024). In our study, the identified peptides carrying a modifying moiety also resided directly in the binding interface (K_SASA_ = 0.5) of two complement factors, C4a and C4b. Figure 4C illustrates the prothrombin protein in complex with coagulation factors Va and Xa; the region carrying modified amino acid residues is located in an unstructured domain residing on the protein globule surface. However, the modification site in this complex is remote from the protein binding interface (K_SASA_ = 1).

No full-atom models are available for three proteins (APOB, TTR, and NRAP) in PDB. The lack of structural data on these proteins in the PDB and AlphaFold databases makes it difficult to map the phosphorylation site with respect to the interface of binding to the partner protein. However, potential partners (presented in Table 7) were identified for the remaining proteins.

Structural analysis of proteins associated with development of diseases (primarily, cancer) is interesting for researchers (e.g., a recent study of the occurrence of α-helix- and β-strand-carrying structural domains in proteins encoded by over- and underexpressed genes) for 21 cancer types. The authors called this study “pan-cancer structurome” and demonstrated an increased amount of β-sandwich domains and insufficient amount of α-helices in proteins (Medvedev et al., 2023).

## 4 Discussion

Kidney diseases are currently extremely common. Although there exists a broad variety of kidney pathologies according to the ICD-10 classification, the literature suggests rather similar clinical presentations of disease development. Moreover, some studies attest to the “mimicry” of kidney diseases (Tseng, 2015; Lu et al., 2021; Lai et al., 2019) and malignant transformation of benign neoplasms (Jiang et al., 2015; Yu et al., 2017). These factors impede diagnosis and timely choice of an efficient treatment option. Histological examination is undoubtedly a potent clinical diagnosis tool providing an unambiguous answer to the question about the neoplasm nature. However, histology cannot be viewed from the standpoint of a routine diagnostic approach, since it is an invasive procedure associated with surgical intervention. It is important to develop novel minimally invasive approaches that would allow one to detect transition from a benign kidney neoplasm to a malignant one. In the present study, we examined changes in protein compositions and proteoforms in three pathologies: kidney cyst, kidney calculus, and kidney cancer. For understanding the molecular differences between the selected pathologies, study subjects involved patients with acquired cysts requiring no surgical intervention for the underlying disease (Bosniak I or II cysts); patients with kidney cancer who predominantly had small-sized tumors and belonged to group T1 (according to the TNM classification); patients in this group had no lymph node metastases (N0). Most patients with kidney calculus were found to have medium- and large-sized stones in the kidney and underwent nephrolithotomy.

We can see that the analyzed pathologies are similar at the protein level. A small group of proteins (n = 9) was revealed, which can be classified as differentially expressed proteins (DEPs) compared to the control group. Because of the specific feature of the selected pathologies, these proteins are involved in activation and implementation of the antibody-dependent response of the immune system (GO:0002250) and simultaneous activation of the complement system via the classical pathway (GO:0006958). Proteins (n = 38, |w| >0.1) making the greatest contribution to segmentation of comparison groups, along with the aforementioned biological processes, are involved in the complement (GO:0006956) and coagulation system (GO: 0050817), lipid transport and metabolism (GO:0006629, GO:0006869), or are acute phase proteins (GO:0006953). Although a relatively modest accuracy (70 or 80%) has been achieved in classifying the comparison groups, close attention to the identified protein factors in combination with clinical observations will possibly improve this result. We have generalized the findings and supplemented them with the available data to reconstruct the potential molecular mechanisms for the involvement of proteins identified in this study.

### 4.1 The complement system

**FIGURE 5:**
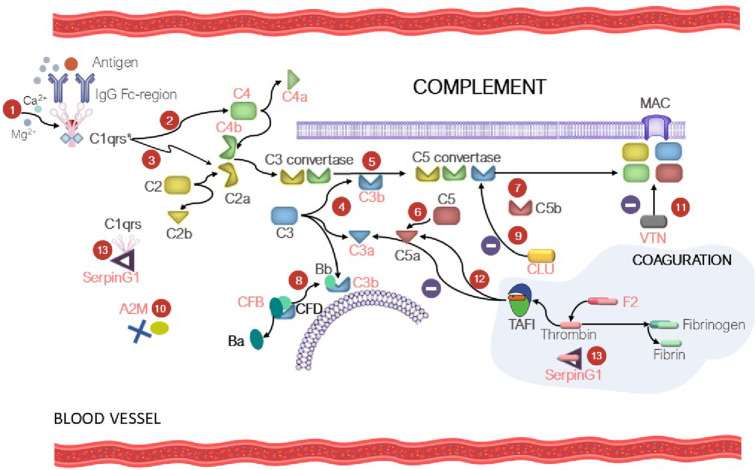
At the first step (1), C1 is bound to the Fc region of the immune complex (IgG-Ag), resulting in С1qrs* activation. At the second step (2), the activated C1 factor cleaves C4 factor yielding C4a (circulating factor) and C4b (exposure to reactive thioester). The C4b fragment binds to the membrane; the C4a fragment becomes circulating. At the third step (3), С1qrs* cleaves C2 into C2b (circulating factor) and C2a (exposure to reactive thioester), which binds to C4a to give rise to the C4b–C2a complex. The C4b-C2a complex is a C3 convertase, which cleaves the C3 factor to C3a (circulating factor) and C3b (exposure to reactive thioester) (4). As a result, the fifth step (5) involves formation of C5 convertase (the C4b-C2a-C3b complex), which participates in cleavage of factor C5 (6) into C5a (circulating factor) and C5b (exposure to reactive thioester) and formation of the membrane attack complex (MAC) (7). In turn, CFD causes cleavage of CFB into factor Ba (circulating factor) and Bb, which binds to C3b to form C3 convertase and then C5 convertase (the alternative pathway) (8). Clusterin (CLU) prevents formation of MAC (9). The tetrameric A2M thioster-containing protein (identically to C3 and C4) entraps active proteases (10). Vitronectin (VTN) is a ligand of factor C9 and modulates its activity (11). The complement and coagulation system crosstalk is mediated by thrombin-activatable fibrinolysis inhibitor (TAFI), which inactivates C3a and C5a in a negative feedback loop (12). SerpinG1 is involved in regulation of the enzyme cascade of the complement and coagulation system through formation of the C1/SerpinG1 and thrombin/G1 complexes (13). Protein factors that were identified in our study are shown in red.

The complement system is the key part of the immunity. Disbalancing of activation regulation of this system may cause inflammation and further development of different diseases (Lin et al., 2018). Patients with various tumor pathologies have increased expression of some complement factors in the classical complement pathway, which contribute to immune surveillance through cytotoxicity and lysis of tumor cells (Afshar-Kharghan, 2017). However, overexpression of the membrane and soluble forms of inhibitors of complement components was observed in the case of solid tumors, protecting the tumor against complement-mediated lysis (Reese et al., 2020). Interestingly, our study revealed increased levels of proteins of the C3/C5 axis, namely, CFB (FC = 1.7, *p* = 0.004), and C3 (FC = 1.3, uncertain value), C4B (FC = 1.5, uncertain value), Clu (FC = 1.4, *p* = 0.065), which is consistent with the literature data on prognostic significance of these factors in human kidney cancer (Xi et al., 2016; Netti et al., 2021; Reese et al., 2020). Proteins CFB, С3, C4A, and C4B are associated with the development of kidney diseases (see Supplementary Table S5). Complement activation may induce or promote glomerular injury in multiple kidney diseases (Vivarelli et al., 2024) (Figure 5). However, conspicuous is the fact that the role of the axis in oncogenesis is rather ambiguous, since a number of studies have demonstrated that tumor grows under C3/C5 activation conditions through recruitment and activation of myeloid suppressor cells in tumors (Reis et al., 2018; Pio et al., 2013; Macor et al., 2018; Berraondo et al., 2016). Acute-phase protein alpha-2-macroglobulin (A2M) is another circulating component potentially associated with the C3/C5 axis of complement activation (the classical pathway). It is a multi-functional high-molecular-weight homotetrameric glycoprotein interacting with proteinases, which induces conformational changes in the monomer molecules and modulates their function (Arimura and Funabiki, 2021; Lagrange et al., 2022). The inhibitory mechanism of A2M involves formation of a tetrameric structure around active proteases and physically hampers the interaction between proteases and substrates (the so-called snap-trap mechanism) (Vandooren and Itoh, 2021a). There are parallels between functioning of C3 and C4 factors and A2M. The pivotal element of the system is the cleavage of C3 and C4 factors followed by exposure of internal thioester binding neighboring glycoproteins (Vandooren and Itoh, 2021a). Similar to C3 and C4, A2M undergoes proteolysis and conformational changes to expose internal thioester that binds and entraps active protease (Vandooren and Itoh, 2021b). Along with C3 and C4, A2M is classified as a member of the thioester-containing protein (TEP) family (Vandooren and Itoh, 2021a). All these factors are eventually involved in formation of the membrane attack complex (MAC), which leads to cell lysis and forces cells release inflammatory cytokines. MAC formation is regulated (namely, inhibited) by clusterin protein, which is essential for protecting own cells against being attacked by the MAC during complement activation (Lin et al., 2018).

Protein serine proteinase complement factor B (CFB) is involved in complement activation (Riihilä et al., 2019). CFB binds to C3b and forms the C3bB complex, participates in formation of C3 convertase and further C5 convertase, acting as an activator of the complement system via the alternative pathway (Figure 5). Dysregulation of these processes may lead to hypercoagulation and thrombosis development. Hyperactivation of the alternative complement pathway with involvement of CFB results in the development of C3 glomerulopathy, which leads to accumulation of the C3 component and glomerular injury. Vitronectin (VTN, FC = 1.3, uncertain value), whose expression is upregulated during complement activation (Ruiz-Molina et al., 2023) is a defense protein and an inhibitor of complement activation via the alternative pathway (Figure 5).

### 4.2 The coagulation system

The proteolytic cascades (the immune system, the complement and the coagulation system) are finely mutually balanced by a network of regulatory factors. The complement–coagulation crosstalk can influence activation, amplification and regulatory functions in both systems (Heurich and McCluskey, 2023). In our study, we noted that proteins of the coagulation system (fibrinogen chain A, B and G (FGA, FGB, FGG, respectively)), as well as phosphorylated prothrombin (F2), contribute to segmentation of the comparison groups. Figure 5 shows the potential mechanism of the crosstalk between the coagulation system and complement factors, the so-called “coagulo-complementome”. Prothrombin and thrombin-activatable fibrinolysis inhibitor (TAFI) mediate this interaction (Danckwardt et al., 2013). Blood clotting factors play an important role in cancer biology (Bauer et al., 2022). Coagulation abnormalities are common among cancer patients who have an increased procoagulant activity due to the release of tumor-derived procoagulants into the bloodstream (Danckwardt et al., 2013). Elevated plasma levels of fibrinogen in patients with renal cell carcinoma were reported in the literature (Selby et al., 2018; Chen et al., 2024; Tupikowski et al., 2023; Wang et al., 2020).

Plasma protease C1 inhibitor (SerpinG1) belongs to the family of serine protease inhibitors and is an important regulator of the complement, coagulation, and fibrinolytic systems. The blood levels of the protease/SerpinG1 complexes (C1/SerpinG1, Thrombin/SerpinG1) are proportional to the level of activation of these enzyme cascades in blood plasma (Kajdácsi et al., 2020) (Figure 5). In our study, we observed that the SerpinG1 level in renal patients was moderately increased (FC = 1.5, *p* = 0.003). The “coagulo-complementome” factors show great promise as therapeutic targets and diagnostic factors (Danckwardt et al., 2013).

#### 4.2.1 Lipid transport and metabolism

Lipid metabolism reprogramming is an obvious sign of cancer. Lipids play a crucial role at the cellular (membrane biosynthesis and energy metabolism) and organ levels (intercellular signaling). Modulation of lipid metabolism allows tumor cells to survive in a nutrient-deprived environment (Heravi et al., 2022). Lipid reprogramming in kidney cancer is related to changes in cholesterol (Wu et al., 2019; Saito et al., 2016; Abduljabbar et al., 2024) and fatty acid (Lv et al., 2019; Wu et al., 2020) metabolism as well as lipolysis reduction (Zhang X. et al., 2017). In our study, we observed significant differences in patients’ weight and BMI for the KD and KC groups vs. the CNTR group. The mean weight of females in the KD group was 87 ± 12.6 kg; in the KC group, 98 ± 12.3 kg; and in the CNTR group, 65.8 ± 6 kg. In males, a similar weight gain was observed in the KD group (86.5 ± 12.7 kg) and KC group (95 ± 10 kg) vs. the CNTR group (68.5 ± 7 kg) (Table 2). According to their BMI, male and female subjects in the KD and KC groups were overweight or had class 1 and class 2 obesity, respectively. Subjects in CNTR either had normal weight or were overweight. Obesity is a risk factor for kidney cancer; elevated body mass index is associated with poor prognosis (Selby et al., 2018). Along with the risk of developing various cancer types, including kidney cancer, overweight is also associated with developing such comorbidities as nonalcoholic fatty liver disease, hypertension, and type 2 diabetes mellitus. Control over hypertension and obesity, as well as quitting smoking, are important measures for primary kidney cancer prevention (Gray and Harris, 2019). At the molecular level, we observed significant elevation of blood levels of triglycerides (FC = 3, *p* = 0.00003) and glucose (FC = 1.4, *p* = 0.002), as well as increased levels of histidine-rich glycoprotein (HRG, FC = 1.7, *p* = 0.02) and serum paraoxonase 1 (PON1, FC = 1.5, uncertain value). Insulin resistance, the key component of the metabolic syndrome, as well as elevation of the triglyceride level are associated with kidney cancer. The assessed triglyceride-glucose index is a prognostic factor of survival in patients with postoperative renal cell carcinoma (Qin et al., 2024).

HRG is a plasma protein playing a crucial role in regulating the immune response, angiogenesis, and hemostasis. It is involved in various biological processes due to its ability to interact with heparin, fibrinogen, platelets, immune cells, and growth factors. Disruption of HRG function is associated with various diseases, including breast cancer and hepatocellular carcinoma. HRG dysfunction may promote tumor tissue growth by disrupting the balance between pro- and anti-angiogenic factors. PON1 enzyme is involved in antitumor response; it inhibits migration, invasion and proliferation of kidney cancer cells and suppresses tumor growth (Li and Yu, 2019). These proteins have a wide range of functions, are involved in tumor progression and antitumor response; in the literature, they are regarded as candidate targets for drugs or prognostic predictors (Johnson et al., 2014; Oiwa et al., 2022; Jiang et al., 2021; Schneider et al., 2016).

#### 4.2.2 Acute phase

There is a well-known association between acute phase proteins (APPs) and oncopathology: a positive correlation between the elevated APP level and an unfavorable clinical outcome has been revealed (Janciauskiene et al., 2021a). In this study, we observed elevated levels of alpha-1-antichymotrypsin (SerpinA3, FC = 1.4, *p* = 0.05), A2M (FC = 1.3, *p* = 0.02), alpha-1-acid glycoprotein 2 (ORM2, FC = 1.4, *p* = 0.02), hemopexin (HP, FC = 4, uncertain value), and ceruloplasmin (CP, FC = 1.3, *p* = 0.04). These proteins are probably associated with the development of different oncopathologies and are viewed in the literature as candidate biomarkers for cancer prognosis and diagnosis of complications of cancer treatment (Janciauskiene et al., 2021a; Lu et al., 2016; Han et al., 2017; Ohbatake et al., 2016; Janciauskiene et al., 2021b). SerpinA3 is a serine protease inhibitor involved in regulation of inflammation and proteolytic activity. In the context of oncopathology, this protein may modulate the tumor microenvironment by inhibiting proteases involved in extracellular matrix degradation. SerpinA3, as well as A2M, alpha-1-acid glycoprotein 2, are considered candidate biomarkers of various cancers, including kidney cancer (Jin et al., 2022; Aibara et al., 2021). Hemopexin is also a circulating protein that plays an important role in protecting the body against heme toxicity during hemolysis. In patients with acute kidney injury, HP compensates for oxidative stress, inflammation, and apoptosis of renal tubular cells as a result of heme accumulation. However, HP deficiency may increase the risk of acute kidney failure progression and development of secondary damage such as diabetic nephropathy. ORM2 is a plasma protein playing a crucial role in regulation of inflammation and immune response. Copper-binding protein CP (ceruloplasmin) is involved in iron and copper metabolism as well as the antioxidant defense of the body. The protein is involved in regulation of redox processes, including oxidation of Fe^2+^ to Fe³^+^, which is required for iron binding to transferrin. Disturbances in ceruloplasmin expression or function are associated with a number of diseases, including aceruloplasminemia, cirrhosis, and Parkinson’s disease. IGHV4-59 (FC = 1.6, *p* = 0.005) is the gene encoding one of immunoglobulin heavy chain variable regions and being involved in immunoglobulin-mediated immune response. The IGHV4-59 gene is engaged in the development of such pathologies as celiac disease and type 2 diabetes mellitus.

#### 4.2.3 Modified proteoforms

Identification of disease-specific proteoforms is important for understanding the nature of oncopathology. There currently is lack of research into identification of modified proteoforms in patients with kidney cancer. In this study, we focused on phosphorylated forms of proteins involved in regulation of the complement system and protein phosphorylation: ApoB, CFB, C4A/C4B, TTR, and NRAP. Structural analysis was conducted for proteins with the annotated 3D structure in the open-source PDB (https://rcsb.org) and AlphaFold (https://alphafold.ebi.ac.uk/) databases, which showed that the modifying moiety resides in β structure-containing structural domains of the ribbon, sandwich, and β-barrel types (according to the CATH database, https://www.cathdb.info/). In nature, these structural domains are involved in organization of protein- or ligand-binding functional domains. Interestingly, the phosphorylation site in the detected proteins is solvent-oriented. An analysis of structural complexes of the identified proteoforms with native partners demonstrated that the modification site can be mapped directly at the interface of binding to the partner or immediately adjacent to it. Phosphorylation often leads to modulation of protein function both in normal and pathological conditions. The location of the modification site at the protein binding interface is indirectly indicative of changes in complex stability (Nikolsky et al., 2023). In the literature, these modification variants are found in various oncopathologies. Thus, according to the PhosphoSitePlus data (Hornbeck et al., 2019), the identified modification variants ApoB 1287-PTR/1288-TPO and TTR 25-TPO were found in biosamples collected from patients with T-cell leukemia; CFB phosphorylation variants were annotated in patients with gastric carcinoma; C4A/C4B and F2 proteins phosphorylated at other sites were identified in breast, ovarian, stomach, head/neck, and colorectal cancers.

Hence, proteins presented in this study can potentially be associated with the development of kidney diseases; it is promising to conduct further analysis of their medical significance with a larger sample size and number of comparison groups. The results of analyzing the association between the identified proteins and development of various diseases and disease groups in accordance with the ICD-10 classification using the Diseases database (Disease–gene associations mined from literature) (Pletscher-Frankild et al., 2015) are reported in Supplementary Table S5. This analysis revealed that proteins described in our study are most commonly mentioned in the literature in the context of developing cancer, diabetes mellitus, kidney diseases, and hypertension.

## 5 Conclusion

This study addresses the features of the protein profile of plasma samples collected from patients with kidney diseases. During the study, we conducted proteomic analysis of blood samples in three comparison groups of study subjects: those with verified diagnosis of kidney calculus, kidney cyst or kidney cancer as well as conditionally healthy volunteers. Blood chemistry parameters in all three groups of study subjects (KC, KD and CNTR) were analyzed at the first stage of the study. Blood chemistry parameters in all the groups of study subjects were normal. Plasma proteins were analyzed at the next stage. DEPs whose blood level was found to be elevated with respect to that in the control group were identified. Hemoglobin subunit beta was found to increase most significantly: its level rose more than twofold (*p* = 0.08) in the KD group and more than fourfold (р = 0.0008) in the KC group.

We have identified proteins contributing most significantly to pathology development, which allowed us to classify patients with analyzed pathologies into groups. CFB, SERPINA3, HPX, HRG, SERPING1, HBB, ORM2, and CP proteins associated with the development of kidney pathologies have been proposed. Important biological processes have been described, and probable signaling pathways of pathology development involving the identified proteins have been elucidated. The paper summarizes important biological processes involving the identified proteins: classical antibody-mediated complement activation (R-HSA-173623, n = 46); FCGR activation (R-HSA-2029481, n = 45); as well as binding and uptake of ligands by scavenger receptors (R-HSA-2173782, n = 54).

The third stage of the study involved searching for and analyzing phosphorylated proteoforms. Phosphorylated proteoforms (CFB, C4A/C4B, F2, APOB, TTR, and NRAP) have been detected in the studied blood samples. Phosphorylated proteoforms specific to groups of patients with kidney diseases were analyzed. PTM location in protein was determined: its coordinate, the secondary structure/structural motif, the functional domain, and the potential role in formation of complexes with native partners. The effect of phosphorylation on protein geometry (changes in solvent-accessible surface area; the number of hydrophobic interactions and hydrogen bonds between the PTM-carrying peptide and the remaining protein portion) was analyzed. It has been demonstrated that peptides carrying modified amino acid residues can reside at the interface of binding to the partner protein or near the binding site. An analysis of modified proteins shows synergism in the pathogenesis of selected disease groups.

It has been demonstrated that patients with kidney pathologies can be classified into groups with an accuracy of (70–80)%. Larger size of comparison groups will enable more clear segmentation of the analyzed pathologies and, therefore, allow one to find a faster solution to the problem of early diagnosis of a particular kidney disease in practical medicine.

## Data Availability

The data presented in the study are deposited in the ProteomeXchange Consortium via the PRIDE repository, accession number PXD051799.
