# Supplementary material for: Plasma proteome fingerprint in kidney diseases
Source: Front Mol Biosci. 2025 Jan 17;11:1494779. doi: 10.3389/fmolb.2024.1494779 (PMC11782039; doi:10.3389/fmolb.2024.1494779)
Supplement: Supplementary file 1 [file DataSheet2.pdf]

| UniProt ID | Gene | Protein name | *Disease | **ICD-10 | Name |
|---|---|---|---|---|---|
| P00751 | CFB | Complement factor B | Atypical hemolytic-uremic syndrome | D59.3 | Haemolytic-uraemic syndrome |
| | | | Kidney disease | N28.9 | Disorder of kidney and ureter, unspecified |
| | | | Thrombocytopenia | D69.6 | Thrombocytopenia, unspecified |
| | | | Hypertension | I10-I15 | Hypertensive diseases |
| | | | C3 glomerulopathy | N03.5 | Glomerulonefrite mesangiocapillare diffusa |
| | | | Age-related macular degeneration | H35 | Other retinal disorders |
| P01024 | C3 | Complement C3 | Atypical hemolytic-uremic syndrome | D59.3 | Haemolytic-uraemic syndrome |
| | | | Kidney disease | N28.9 | Disorder of kidney and ureter, unspecified |
| | | | Complement component 3 deficiency | | |
| P0C0L5 | C4B | Complement C4-B | Atypical hemolytic-uremic syndrome | D59.3 | Haemolytic-uraemic syndrome |
| | | | Kidney disease | N28.9 | Disorder of kidney and ureter, unspecified |
| | | | Complement component 3 deficiency | | |
| P0C0L4 | C4A | Complement C4-A | Atypical hemolytic-uremic syndrome | D59.3 | Haemolytic-uraemic syndrome |
| | | | Kidney disease | N28.9 | Disorder of kidney and ureter, unspecified |
| | | | Complement component 3 deficiency | | |
| P01023 | A2M | Alpha-2-macroglobulin | Cancer | C00-C97 | Malignant neoplasms |
| | | | Kidney disease | N28.9 | Disorder of kidney and ureter, unspecified |
| | | | Thrombosis | N28.0 | Ischaemia and infarction of kidney |
| P00751 | CFB | Complement factor B | Kidney disease | N28.9 | Disorder of kidney and ureter, unspecified |
| | | | Hypertension | I10-I15 | Hypertensive diseases |
| | | | Anemia | D50-D89 | Diseases of the blood and blood-forming organs and certain disorders involving the immune mechanism |
| | | | Atypical hemolytic-uremic syndrome | D59.3 | Haemolytic-uraemic syndrome |
| P04004 | VTN | Vitronectin | Cancer | C00-C97 | Malignant neoplasms |
| | | | Hematopoietic system disease | | |
| | | | Thrombosis | I82.9 | Acute embolism and thrombosis |
| P02671 | FGA | Fibrinogen alpha chain | Anemia | D50-D89 | Diseases of the blood and blood-forming organs and certain disorders involving the immune mechanism |
| | | | Cancer | C00-C97 | Malignant neoplasms |
| | | | Malaria | B54 | Unspecified malaria |
| P02675 | FGB | Fibrinogen beta chain | Thrombosis | I82 | Other venous embolism and thrombosis |
| | | | Hypertension | I10-I15 | Hypertensive diseases |
| | | | Cancer | C00-C97 | Malignant neoplasms |
| P02679 | FGG | Fibrinogen gamma chain | Thrombosis | I82 | Other venous embolism and thrombosis |
| | | | Cancer | C00-C97 | Malignant neoplasms |
| P68871 | HBB | Hemoglobin subunit beta | Anemia | D50-D89 | Diseases of the blood and blood-forming organs and certain disorders involving the immune mechanism |
| | | | Cancer | C00-C97 | Malignant neoplasms |
| P00734 | F2 | Prothrombin | Thrombosis | I82 | Other venous embolism and thrombosis |
| | | | Hypertension | I10-I15 | Hypertensive diseases |
| P05155 | SERPING1 | Plasma protease C1 inhibitor | C1 inhibitor deficiency | D84.1 | Defects in the complement system |
| | | | Kidney disease | N28.9 | Disorder of kidney and ureter, unspecified |
| | | | Thrombosis | I82 | Other venous embolism and thrombosis |
| | | | Hereditary angioedema | D80-D89 | Certain disorders involving the immune mechanism |
| | | | Neonatal lung disease | P19-P29 | Respiratory and cardiovascular disorders specific to the perinatal period |
| P01011 | SERPINA3 | Alpha-1-antichymotrypsin | Cancer | C00-C97 | Malignant neoplasms |
| | | | Prostate disease | N51.0 | Disorders of prostate in diseases classified elsewhere |
| | | | Alpha 1-antitrypsin deficiency | E88.0 | Disorders of plasma-protein metabolism, not elsewhere classified |
| | | | Malignant tumor of breast | C50 | Malignant neoplasms of breast |
| P19652 | ORM2 | Alpha-1-acid glycoprotein 2 | Cancer | C00-C97 | Malignant neoplasms |
| | | | Kidney disease | N28.9 | Disorder of kidney and ureter, unspecified |
| | | | Male infertility with asthenoteratozoospermia | N46 | Male infertility |
| | | | Ciliary dyskinesia | Q34 | Other congenital malformations of respiratory system |
| P00738 | HP | Haptoglobin | Anemia | D50-D89 | Diseases of the blood and blood-forming organs and certain disorders involving the immune mechanism |
| | | | Cancer | C00-C97 | Malignant neoplasms |
| | | | Kidney disease | N28.9 | Disorder of kidney and ureter, unspecified |
| P00450 | CP | Ceruloplasmin | Aceruloplasminemia | | |
| | | | Hemochromatosis | E.83.1 | Disorders of iron metabolism |
| | | | Cirrhosis, Hepatic | K70-K77 | Diseases of liver |
| | | | Parkinson Disease | G20 | Parkinson's Disease |
| P02765 | AHSG | Alpha-2-HS-glycoprotein | Chronic kidney disease | N18 | Chronic kidney disease |
| | | | Type 2 diabetes mellitus | E11 | Type 2 diabetes mellitus |
| | | | Obesity | E36-E68 | Obesity and other hyperalimentation |
| P19823 | ITIH2 | Inter-alpha-trypsin inhibitor heavy | Cancer | C00-C97 | Malignant neoplasms |
| | | | Autoimmune disease | | |
| Q14624 | ITIH4 | Inter-alpha-trypsin inhibitor heavy | Cancer | C00-C97 | Malignant neoplasms |
| P27169 | PON1 | Serum paraoxonase/arylesterase 1 | Lipid metabolism disorder | E78.9 | Disorder of lipoprotein metabolism, unspecified |
| | | | Kidney disease | N28.9 | Disorder of kidney and ureter, unspecified |
| P02787 | TF | Serotransferrin | Cancer | C00-C97 | Malignant neoplasms |
| | | | Diabetes mellitus | E10-E14 | Diabetes mellitus |
| P02790 | HPX | Hemopexin | Cancer | C00-C97 | Malignant neoplasms |
| | | | Kidney disease | N28.9 | Disorder of kidney and ureter, unspecified |
| P04196 | HRG | Histidine-rich glycoprotein | Cancer | C00-C97 | Malignant neoplasms |
| P01825 | IGHV4-59 | Ig | Cancer | C00-C97 | Malignant neoplasms |
| | | | Celiac Disease | K90 | Intestinal malabsorption |
| | | | Type 2 diabetes | E11 | Type 2 diabetes mellitus |
| Serotransferrin | | TF | Cancer | C00-C97 | Malignant neoplasms |

***https://diseases.jensenlab.org/Search**

****ICD-10 Version:2019**
