# Supplementary material for: Plasma proteome fingerprint in kidney diseases
Source: Front Mol Biosci. 2025 Jan 17;11:1494779. doi: 10.3389/fmolb.2024.1494779 (PMC11782039; doi:10.3389/fmolb.2024.1494779)
Supplement: Supplementary file 4 [file DataSheet1.pdf]

P00751: LEDSVTYHCSR

CFB Complement factor B

Picture	Description	SASA
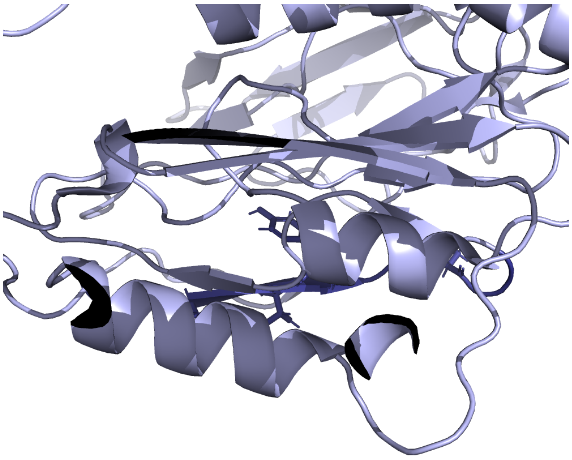	**2OK5_A158_A169**<br><br>Human Complement factor B	extracted SASA: 1868.65478515625<br><br>SASA in chain: 632.904541015625<br><br>SASA in complex: 632.904541015625
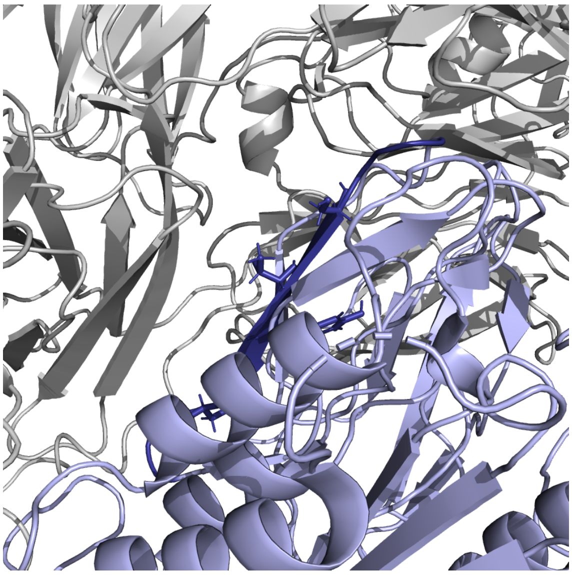	**2XWB_F158_F169**<br><br>Crystal Structure of Complement C3b in complex with Factors B and D<br><br>partners:<br><br>• P01024 Complement C3	extracted SASA: 1880.1937255859373<br><br>SASA in chain: 682.3202514648438<br><br>SASA in complex: 368.4106140136719
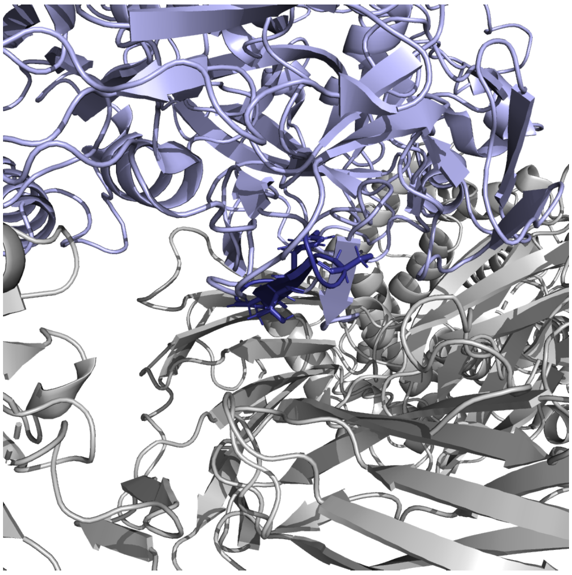	**2XWB_H158_H169**<br><br>Crystal Structure of Complement C3b in complex with Factors B and D<br><br>partners:<br><br>• P01024 Complement C3	extracted SASA: 1870.9134521484373<br><br>SASA in chain: 685.4497680664062<br><br>SASA in complex: 380.173828125

Picture	Description	SASA
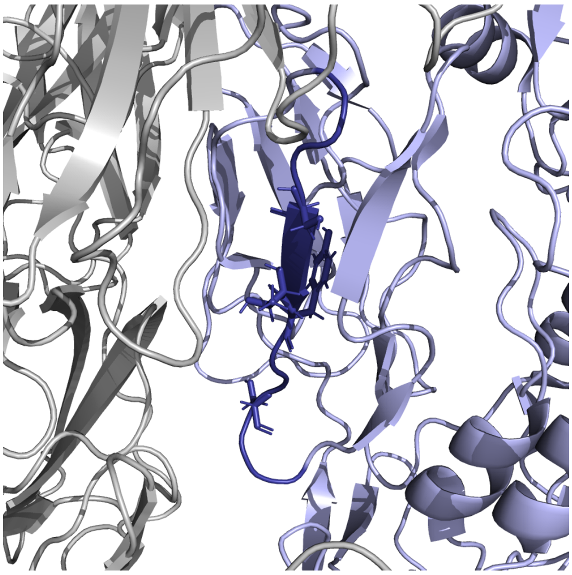	**2XWJ_I158_I169**<br>Crystal Structure of Complement C3b in Complex with Factor B<br>partners:<br>• P01024 Complement C3	extracted SASA: 1880.964599609375<br>SASA in chain: 694.4461059570312<br>SASA in complex: 364.3595581054688
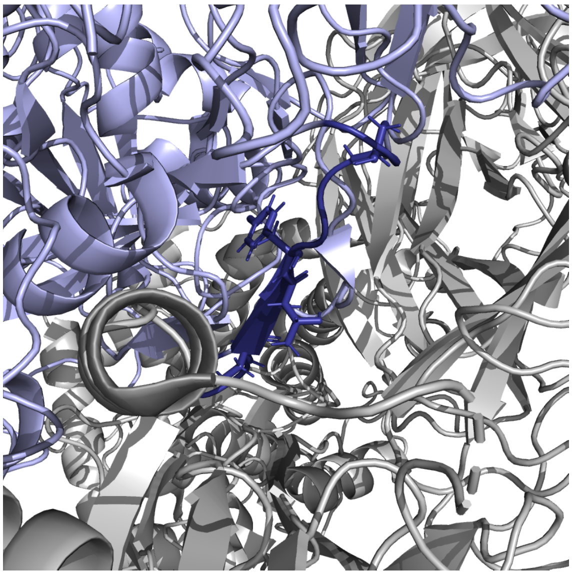	**2XWJ_J158_J169**<br>Crystal Structure of Complement C3b in Complex with Factor B<br>partners:<br>• P01024 Complement C3	extracted SASA: 1866.4263916015625<br>SASA in chain: 773.6297607421875<br>SASA in complex: 437.9132995605469
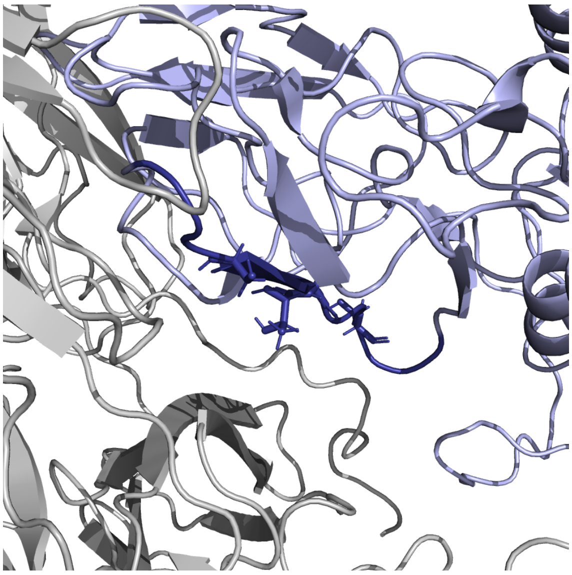	**2XWJ_K158_K169**<br>Crystal Structure of Complement C3b in Complex with Factor B<br>partners:<br>• P01024 Complement C3	extracted SASA: 1864.375<br>SASA in chain: 772.2628173828125<br>SASA in complex: 438.5135192871094

Picture	Description	SASA
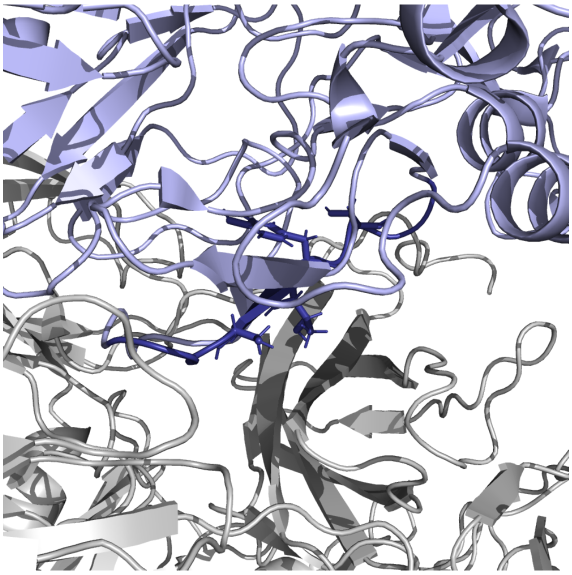	**2XWJ_L158_L169**<br><br>Crystal Structure of Complement C3b in Complex with Factor B<br><br>partners:<br><br>• P01024 Complement C3	extracted SASA: 1886.4871826171875<br><br>SASA in chain: 694.341064453125<br><br>SASA in complex: 358.2356872558594
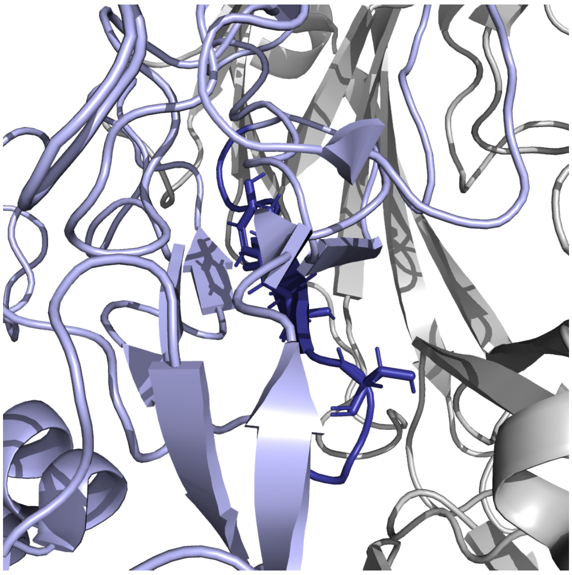	**3HRZ_D158_D169**<br><br>Cobra Venom Factor (CVF) in complex with human factor B<br><br>partners:<br><br>• Q91132 Cobra venom factor	extracted SASA: 1897.62890625<br><br>SASA in chain: 670.1173706054688<br><br>SASA in complex: 371.689453125
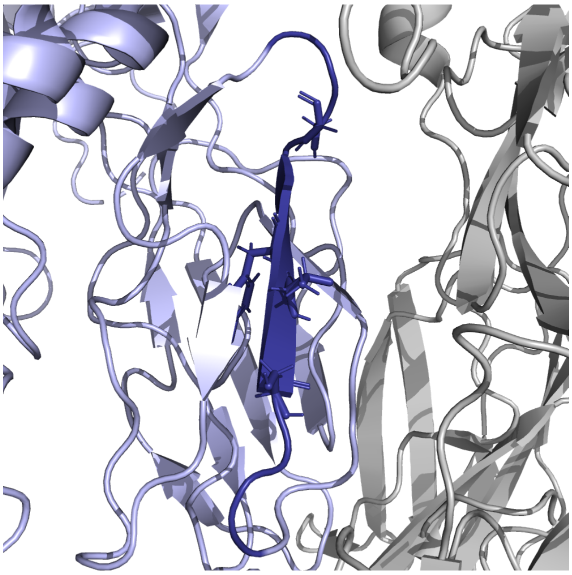	**3HS0_D158_D169**<br><br>Cobra Venom Factor (CVF) in complex with human factor B<br><br>partners:<br><br>• Q91132 Cobra venom factor	extracted SASA: 1875.264892578125<br><br>SASA in chain: 747.3134765625<br><br>SASA in complex: 501.5405578613281

Picture	Description	SASA
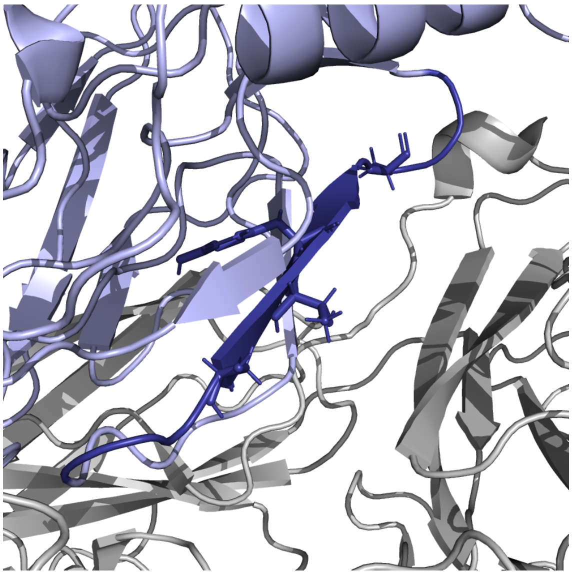	**3HS0_I158_I169**<br><br>Cobra Venom Factor (CVF) in complex with human factor B<br><br>partners:<br><br>• Q91132 Cobra venom factor	extracted SASA: 1875.739501953125<br><br>SASA in chain: 703.6385498046875<br><br>SASA in complex: 445.8577880859375
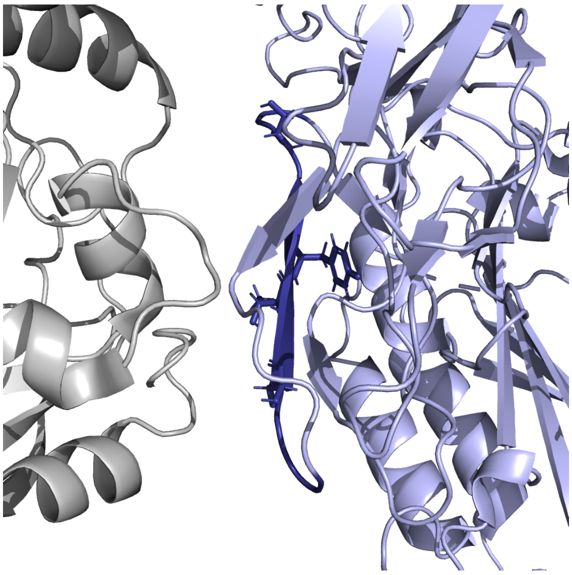	**7JTN_A158_A169**<br><br>Human Complement Factor B Inhibited by a Slow Off-Rate Modified Aptamer of 29 Bases<br><br>partners:<br><br>• P00751 Complement factor B	extracted SASA: 1862.0599365234373<br><br>SASA in chain: 741.3786010742188<br><br>SASA in complex: 628.4021606445312
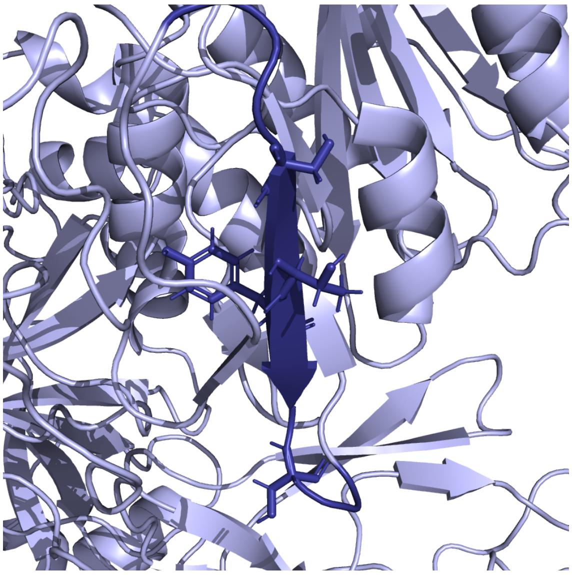	**7JTN_C158_C169**<br><br>Human Complement Factor B Inhibited by a Slow Off-Rate Modified Aptamer of 29 Bases	extracted SASA: 1870.9078369140625<br><br>SASA in chain: 749.2068481445312<br><br>SASA in complex: 672.5419921875

Picture	Description	SASA
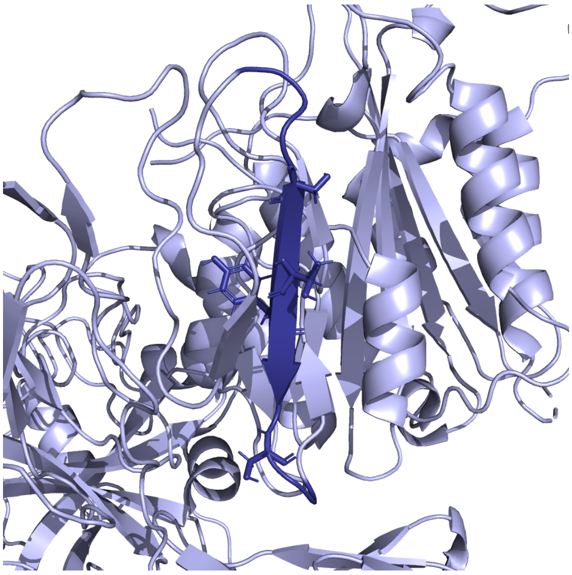	**7JTQ_A158_A169**<br><br>Human Complement Factor B Inhibited by a Slow Off-Rate Modified Aptamer of 31 Bases	extracted SASA: 1852.23193359375<br><br>SASA in chain: 733.3604125976562<br><br>SASA in complex: 659.5974731445312
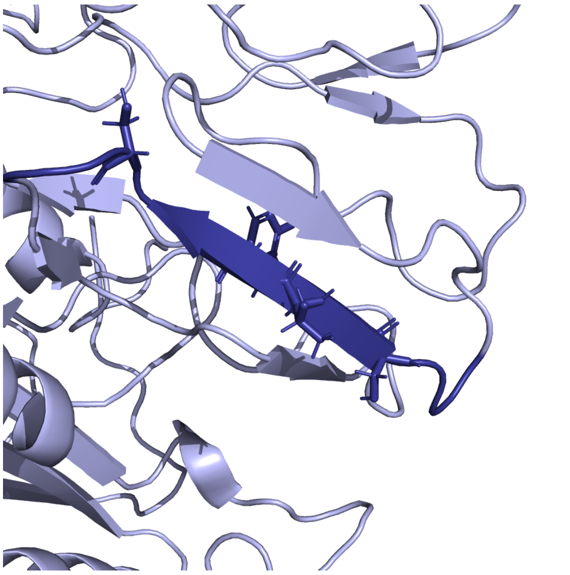	**7JTQ_C158_C169**<br><br>Human Complement Factor B Inhibited by a Slow Off-Rate Modified Aptamer of 31 Bases	extracted SASA: 1858.9561767578125<br><br>SASA in chain: 737.9097900390625<br><br>SASA in complex: 664.3743286132812
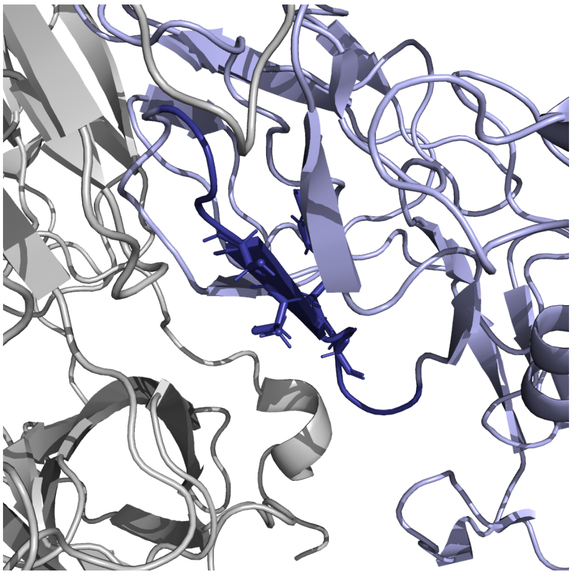	**7NOZ_F183_F194**<br><br>Structure of the nanobody stablized properdin bound alternative pathway proconvertase C3b:FB:FP<br><br>partners:<br><br>• P01024 Complement C3	extracted SASA: 1881.9068603515625<br><br>SASA in chain: 685.1071166992188<br><br>SASA in complex: 367.2398376464844

P0C0L5: TTNIQGINLLFSSR

C4B Complement C4-B

Picture	Description	SASA

Picture	Description	SASA
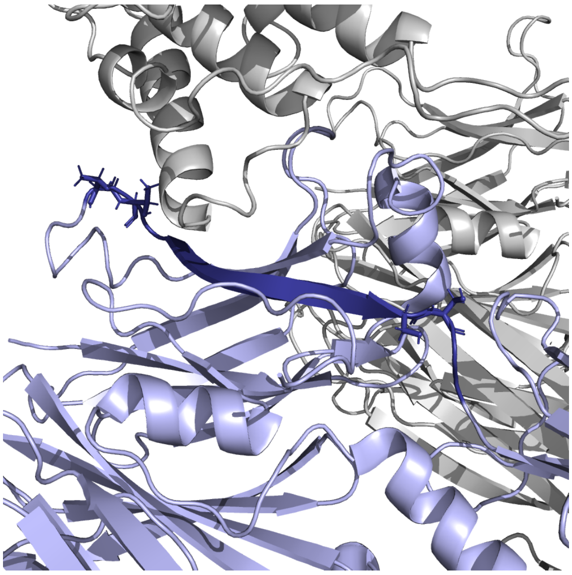	**6YSQ_A124_A138**<br>The hC4Nb8 complement inhibitory nanobody in complex with C4b<br>partners:<br>• P0C0L4 Complement C4-A<br>• P0C0L5 Complement C4-B	extracted SASA: 2454.91845703125<br>SASA in chain: 563.0791015625<br>SASA in complex: 295.2054443359375
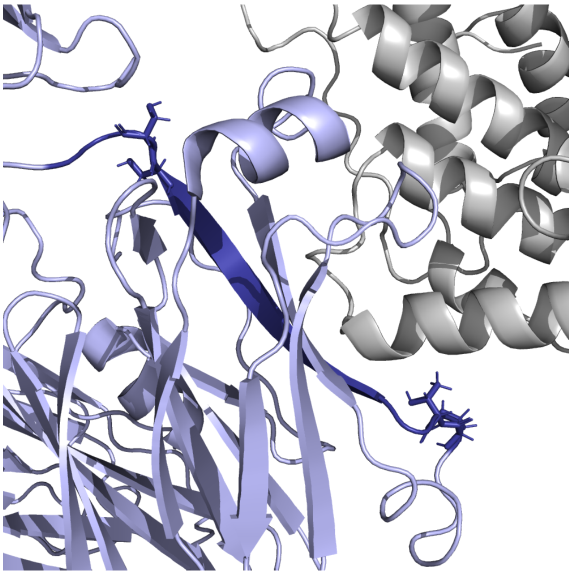	**6YSQ_B124_B138**<br>The hC4Nb8 complement inhibitory nanobody in complex with C4b<br>partners:<br>• P0C0L4 Complement C4-A	extracted SASA: 2457.5947265625<br>SASA in chain: 545.8331909179688<br>SASA in complex: 277.2547302246094

P0C0L5: DFALLSLQVPLK

C4B Complement C4-B

Picture	Description	SASA
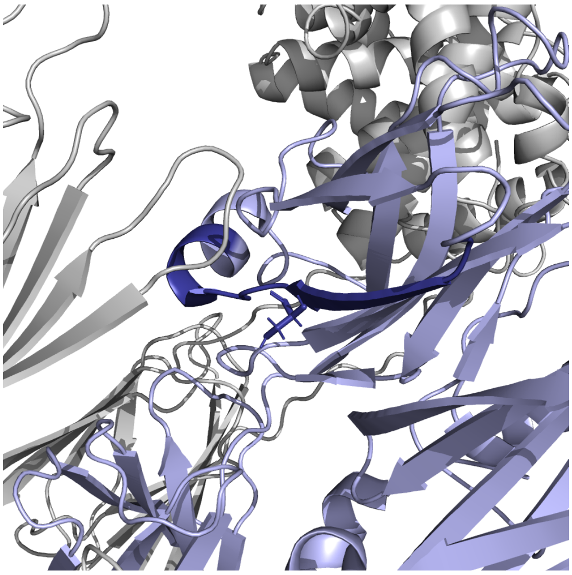	**6YSQ_A81_A93**<br>The hC4Nb8 complement inhibitory nanobody in complex with C4b<br>partners:<br>• P0C0L4 Complement C4-A<br>• P0C0L5 Complement C4-B	extracted SASA: 2024.3126220703125<br>SASA in chain: 665.5379028320312<br>SASA in complex: 605.8429565429688

Picture	Description	SASA
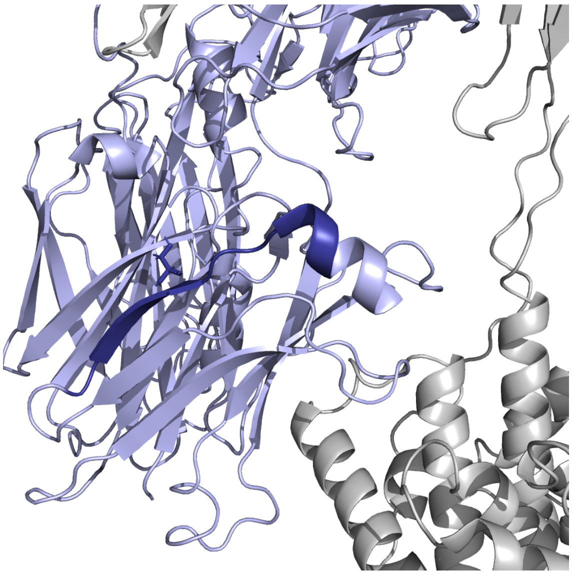	**6YSQ_B81_B93**<br><br>The hC4Nb8 complement inhibitory nanobody in complex with C4b<br><br>partners:<br><br>• P0C0L4 Complement C4-A	extracted SASA: 2034.8778076171875<br><br>SASA in chain: 641.2355346679688<br><br>SASA in complex: 641.2355346679688

P0C0L4: TTNIQGINLLFSSR

C4A Complement C4-A

Picture	Description	SASA
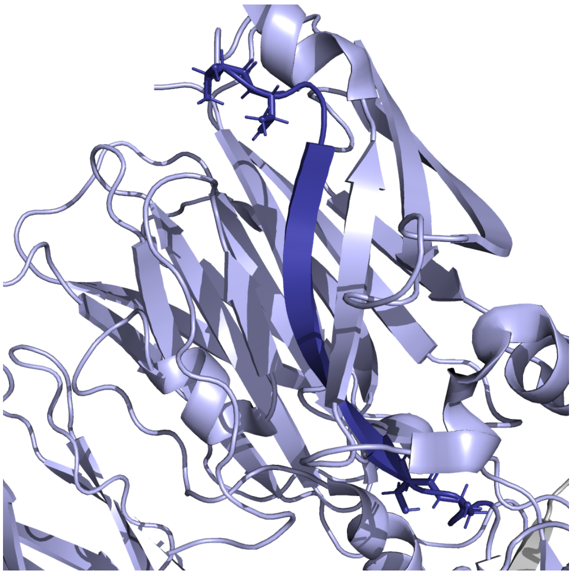	**5JPM_A124_A138**<br><br>Structure of the complex of human complement C4 with MASP-2 rebuilt using iMDFF<br><br>partners:<br><br>• P0C0L4 Complement C4-A	extracted SASA: 2450.076171875<br><br>SASA in chain: 442.7997741699219<br><br>SASA in complex: 442.7997741699219
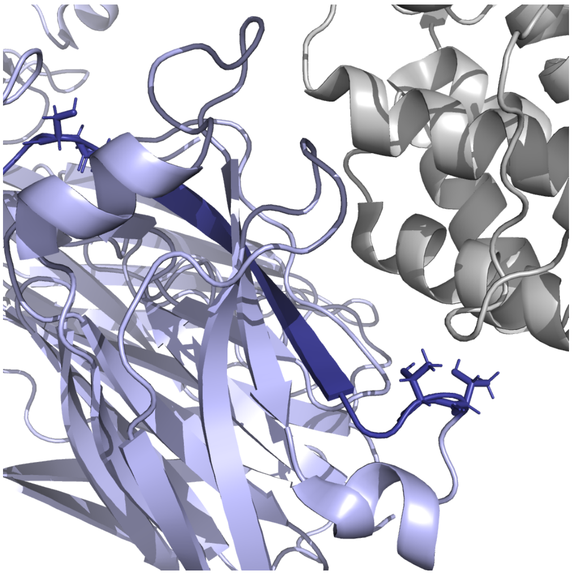	**5JPM_D124_D138**<br><br>Structure of the complex of human complement C4 with MASP-2 rebuilt using iMDFF<br><br>partners:<br><br>• P0C0L4 Complement C4-A	extracted SASA: 2457.68359375<br><br>SASA in chain: 501.7239379882813<br><br>SASA in complex: 329.3780212402344

Picture	Description	SASA
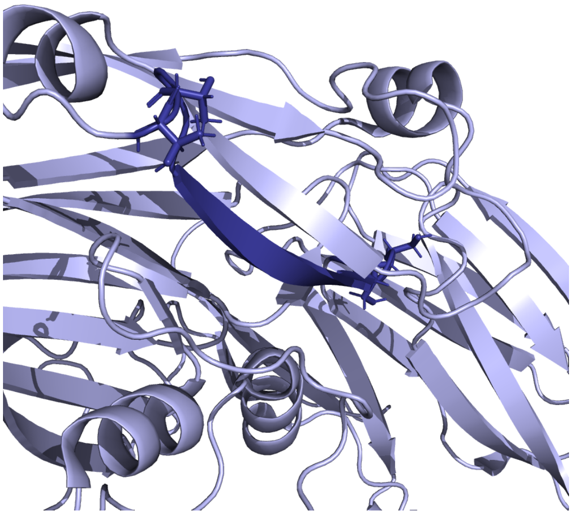	**5JPN_A124_A138**<br>Structure of human complement C4 rebuilt using iMDFF	extracted SASA: 2421.5634765625<br>SASA in chain: 481.9118347167969<br>SASA in complex: 481.9118347167969
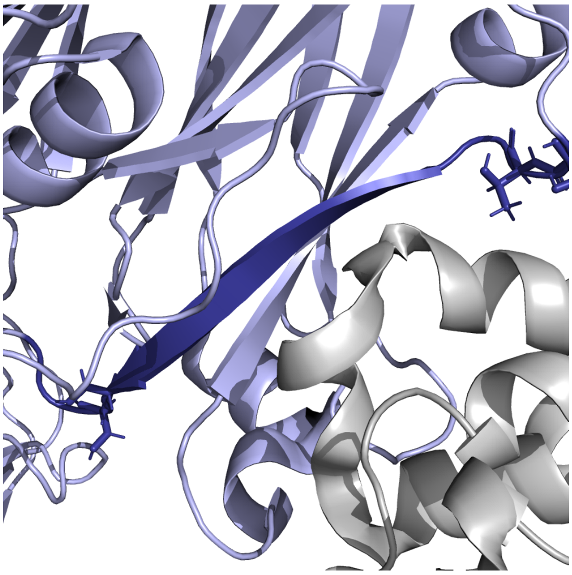	**5JTW_A124_A138**<br>Crystal structure of complement C4b re-refined using iMDFF<br>partners:<br>• P0C0L4 Complement C4-A	extracted SASA: 2438.5791015625<br>SASA in chain: 454.6777648925781<br>SASA in complex: 213.33871459960935
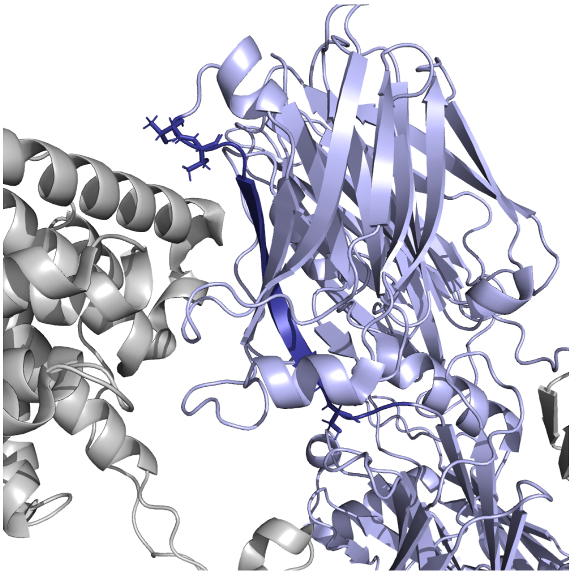	**5JTW_D124_D138**<br>Crystal structure of complement C4b re-refined using iMDFF<br>partners:<br>• P0C0L4 Complement C4-A	extracted SASA: 2454.4521484375<br>SASA in chain: 520.9789428710938<br>SASA in complex: 274.8993835449219

P0C0L4: DFALLSLQVPLK

C4A Complement C4-A

Picture	Description	SASA

Picture	Description	SASA
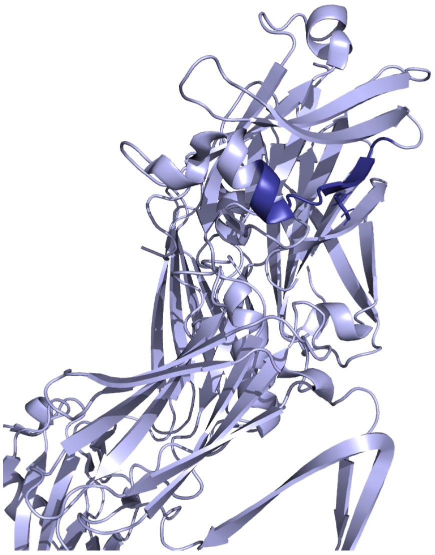	**5JPM_A81_A93**<br><br>Structure of the complex of human complement C4 with MASP-2 rebuilt using iMDFF	extracted SASA: 1983.8359375<br><br>SASA in chain: 673.1837158203125<br><br>SASA in complex: 673.1837158203125
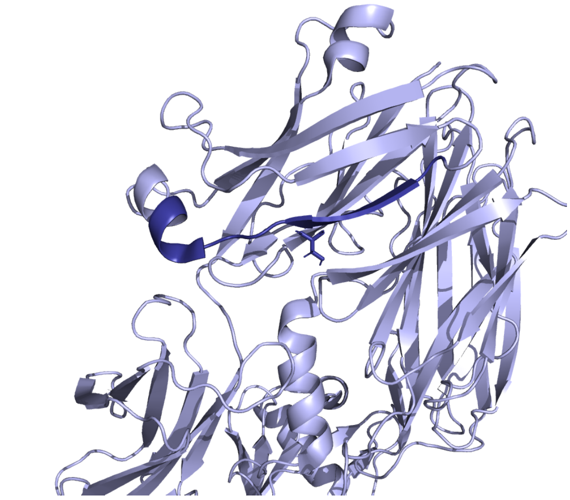	**5JPM_D81_D93**<br><br>Structure of the complex of human complement C4 with MASP-2 rebuilt using iMDFF	extracted SASA: 1985.234130859375<br><br>SASA in chain: 652.4196166992188<br><br>SASA in complex: 652.4196166992188
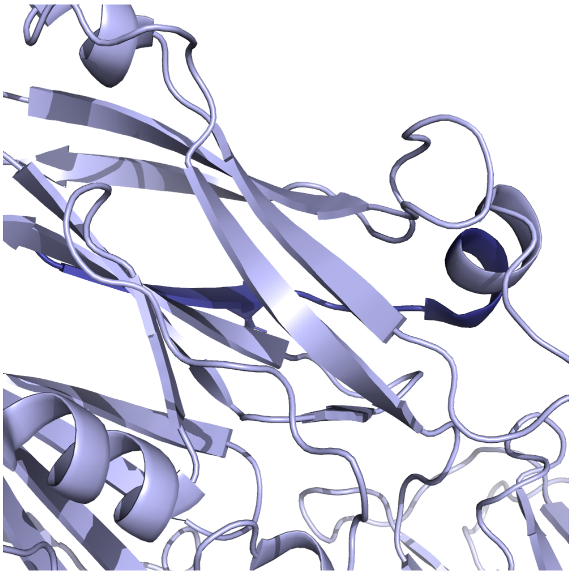	**5JPN_A81_A93**<br><br>Structure of human complement C4 rebuilt using iMDFF	extracted SASA: 1955.5244140625<br><br>SASA in chain: 612.9089965820312<br><br>SASA in complex: 612.9089965820312

Picture	Description	SASA
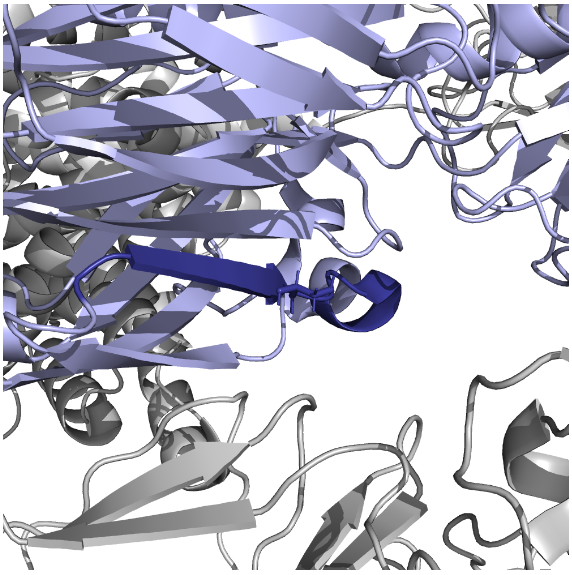	**5JTW_A81_A93**<br>Crystal structure of complement C4b re-refined using iMDFF<br>partners:<br>• P0C0L4 Complement C4-A	extracted SASA: 1990.9693603515625<br>SASA in chain: 623.0714721679688<br>SASA in complex: 569.8309326171875
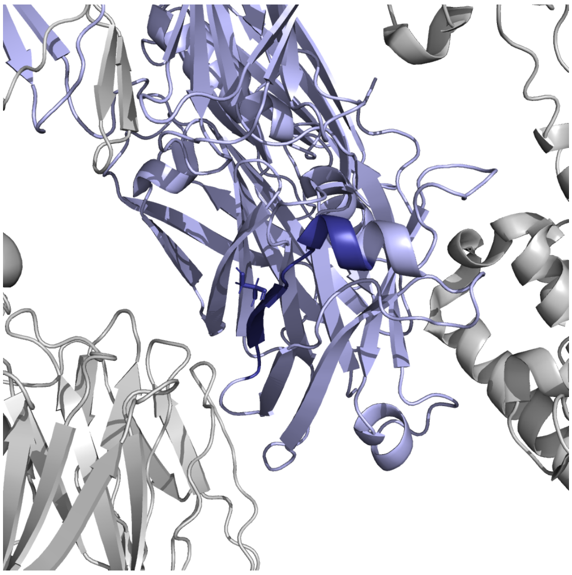	**5JTW_D81_D93**<br>Crystal structure of complement C4b re-refined using iMDFF<br>partners:<br>• P0C0L4 Complement C4-A	extracted SASA: 2004.151611328125<br>SASA in chain: 605.852783203125<br>SASA in complex: 560.7033081054688

P00734: GHVNITR

F2 Prothrombin

Picture	Description	SASA
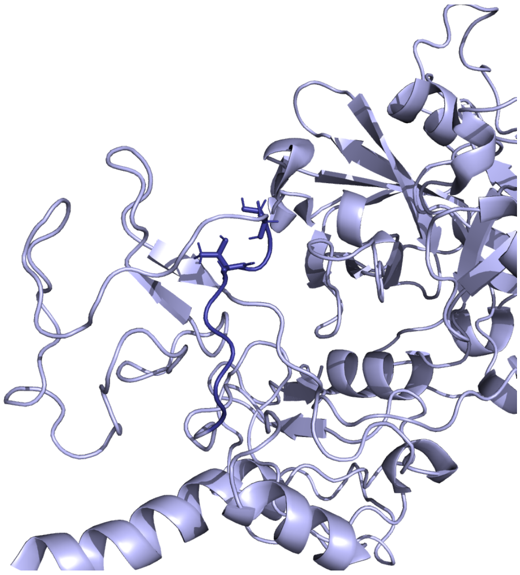	**4NZQ_A75_A82**<br>Crystal structure of Ca2+-free prothrombin deletion mutant residues 146-167	extracted SASA: 1277.5306396484375<br>SASA in chain: 583.910888671875<br>SASA in complex: 583.910888671875

Picture	Description	SASA
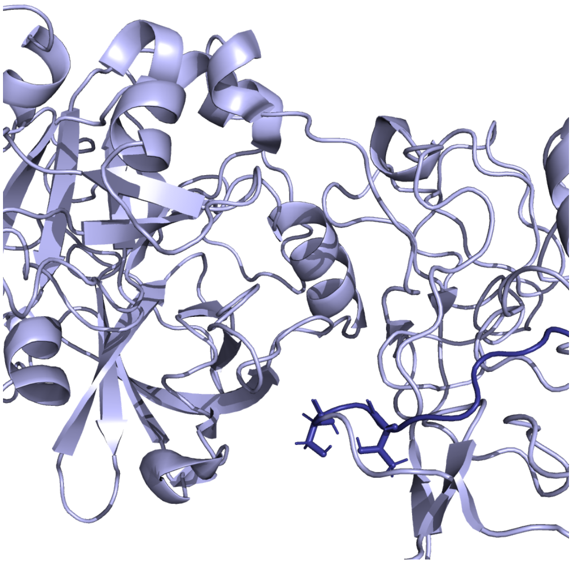	**4O03_A75_A82**<br><br>Crystal structure of Ca2+ bound prothrombin deletion mutant residues 146-167	extracted SASA: 1246.408447265625<br><br>SASA in chain: 594.2027587890625<br><br>SASA in complex: 594.2027587890625
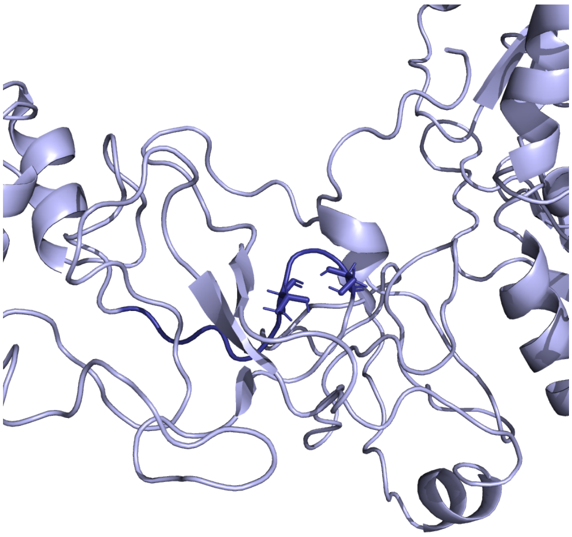	**5EDK_A75_A82**<br><br>Crystal structure of prothrombin deletion mutant residues 146-167 (Form II).	extracted SASA: 1265.042724609375<br><br>SASA in chain: 403.2025451660156<br><br>SASA in complex: 403.2025451660156
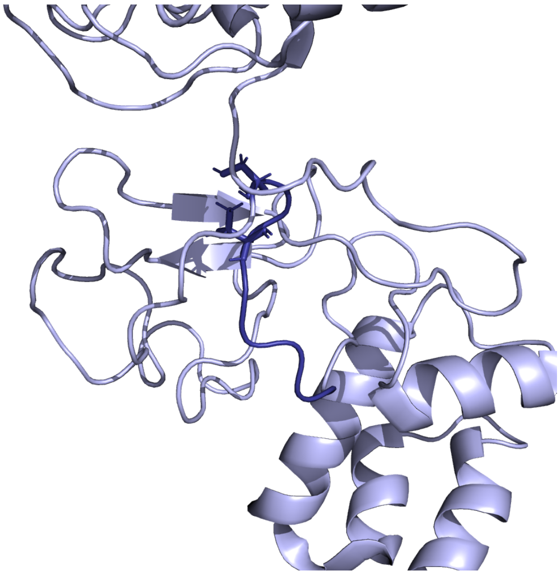	**5EDM_A75_A82**<br><br>Crystal structure of prothrombin deletion mutant residues 154-167 (Form I)	extracted SASA: 1247.0528564453125<br><br>SASA in chain: 359.6258850097656<br><br>SASA in complex: 359.6258850097656

Picture	Description	SASA
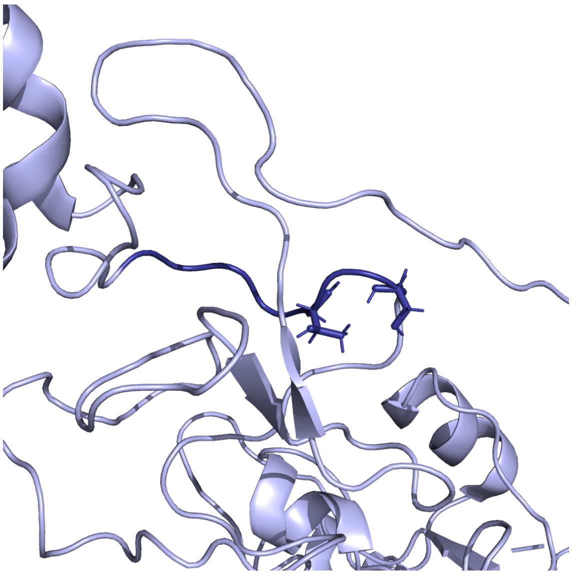	**6BJR_A75_A82**<br><br>Crystal structure of prothrombin mutant S101C/ A470C	extracted SASA: 1249.0582275390625<br><br>SASA in chain: 427.409423828125<br><br>SASA in complex: 427.409423828125
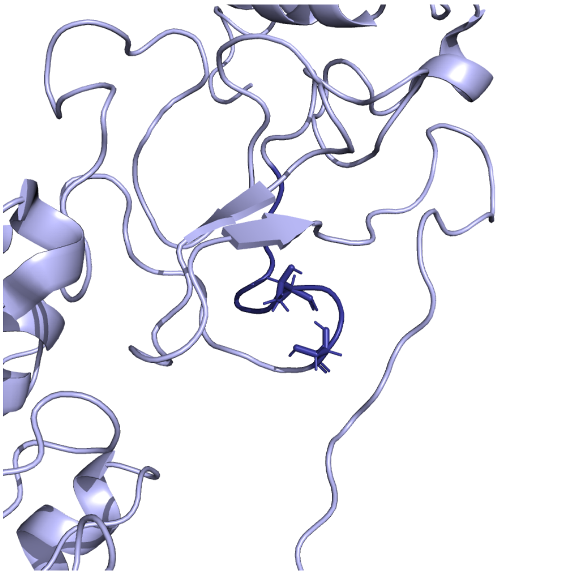	**6C2W_A75_A82**<br><br>Crystal structure of human prothrombin mutant S101C/ A470C	extracted SASA: 1231.1279296875<br><br>SASA in chain: 431.9875183105469<br><br>SASA in complex: 431.9875183105469
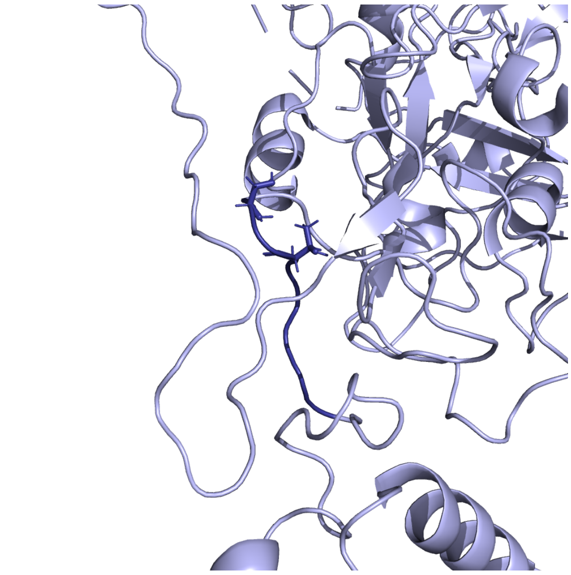	**6C2W_B75_B82**<br><br>Crystal structure of human prothrombin mutant S101C/ A470C	extracted SASA: 1242.1080322265625<br><br>SASA in chain: 442.0482177734375<br><br>SASA in complex: 442.0482177734375

Picture	Description	SASA
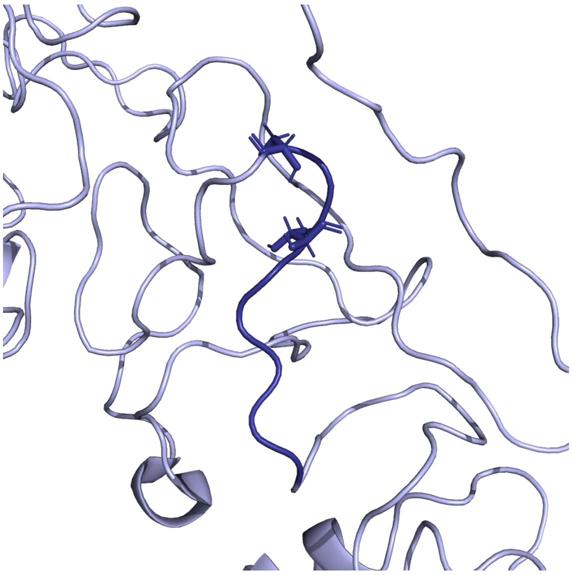	**7TPP_E75_E82**<br><br>Cryo-em structure of human prothrombin:prothrombinase at 4.1 Angstrom resolution	extracted SASA: 1281.1649169921875<br><br>SASA in chain: 512.951171875<br><br>SASA in complex: 512.951171875
